# Long-term real-world PM_2.5_ exposure induces depression-like behaviors in mice by disrupting nuclear factor erythroid 2-related factor 2-mediated astrocyte-to-microglia communication

**DOI:** 10.4103/NRR.NRR-D-24-01469

**Published:** 2025-08-13

**Authors:** Nannan Huang, Weiqing Shi, Cuishuang Dong, Bin Li, Yaohan Wang, Hanqing Chen, Xiaobo Li

**Affiliations:** 1 School of Public Health, Beijing Key Laboratory of Environment and Aging, Capital Medical University, Beijing, China; 2 Jiangsu Provincial Center for Disease Control and Prevention, Nanjing, Jiangsu Province, China; 3 Department of Nutrition & Food Hygiene, School of Public Health, Capital Medical University, Beijing, China

**Keywords:** air pollution, astrocyte-to-microglia communication, depression-like behaviors, fine particulate matter (PM_2.5_), neurotoxicity, nuclear factor erythroid 2-related factor 2, oxidative stress, procyanidins

## Abstract

Long-term exposure to ambient fine particulate matter (PM_2.5_) may increase the risk of neurotoxicity in human populations. However, research studies on the underlying mechanisms of chronic PM_2.5_-induced depression-like behaviors, and potential therapeutical strategies, remain scarce. In the present study, after long-term exposure to real-world PM_2.5_ for 15 weeks, male mice displayed depression-like behaviors, which were revealed using the open field and sucrose preference tests. Mechanistically, chronic PM_2.5_ exposure promoted astrocytic A1 polarization and disrupted reduction–oxidation balance in the mouse hippocampus. Furthermore, PM_2.5_-exposed mice displayed pathological damage to hippocampal neurons as well as the inhibition of nuclear factor erythroid 2-related factor 2 signaling. Astrocytic ablation of nuclear factor erythroid 2-related factor 2 exacerbated PM_2.5_-induced hippocampal neuronal injury in mice via the disruption of astrocyte-to-microglia communication; this finding was confirmed in mice with bilateral and unilateral hippocampal astrocytic *Nfe2l2* knockdown. Importantly, the upregulation of nuclear factor erythroid 2-related factor 2 activation by procyanidin significantly ameliorated PM_2.5_-induced depression-like behaviors through the remodeling of astrocyte-to-microglia communication. Together, our findings shed light on the important role of hippocampal astrocytic nuclear factor erythroid 2-related factor 2 activation for maintaining astrocyte-to-microglia communication, and indicate potential research avenues for therapeutic strategies against PM_2.5_-induced depresson-like behaviors.

## Graphical abstract

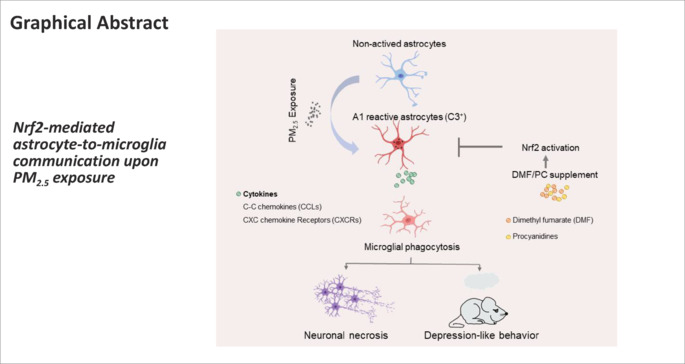

## Introduction

Fine particulate matter (PM_2.5_; diameter of particles: ≤ 2.5 µm) contains a cocktail of organic and inorganic chemicals and represents the main constituent of air pollution in the atmosphere. It is generated from human and natural sources. Increasing epidemiological evidence has demonstrated that chronic exposure to ambient PM_2.5_ is strongly associated with an increased risk of depressive symptoms in both human and animal populations (Buoli et al., 2018; Thomson, 2019; Wu et al., 2024). A systematic review reported an association between long-term PM_2.5_ exposure and depression, with a pooled odds ratio of 1.102 per 10 μg/m^3^ PM_2.5_ increase (Braithwaite et al., 2019); however, the evidence for potential causal associations between real-world PM_2.5_ exposure and depression remains limited.

With the sustained pressure of modern life and work, depression has increased substantially in recent years; it is estimated by the World Health Organization to affect more than 350 million people worldwide by 2030 (Dominiak et al., 2021). Patients with depression exhibit limited psychosocial function and low quality of life. Nonetheless, the pathophysiology of depression remains incompletely understood (Park and Zarate, 2019). Astrocytes have an important role in the homeostasis of synaptic connectivity and plasticity (Heiss et al., 2021). They represent a large glial population in the mammalian brain (Freeman, 2010) and have been applied as a therapeutic target of depression (O’Leary and Mechawar, 2021). Astrocytic markers such as S100 calcium-binding protein B (S100β), glial fibrillary acidic protein (GFAP), gap junction proteins, and membrane channel proteins are often used as markers of minor depression (Joshi and Benerjee, 2018).

A neural microenvironment with reduction–oxidation (redox) imbalance has also been recognized as a vital risk factor for depression (Bhatt et al., 2020). Results from a meta-analysis reported an association between depression accompanied by high oxidative stress and low levels of antioxidant markers (Palta et al., 2014). The central nervous system (CNS) is one of the most vulnerable organs to oxidative stress, which has been postulated to underlie PM_2.5_ exposure-induced neurotoxicity (Ahadullah et al., 2021; Wang et al., 2021). As essential homeostatic cells in the CNS, astrocytes play a crucial role in regulating the balance between oxidative stress generation and antioxidant responses (Chen et al., 2020). In pathological processes, oxidative stress is reportedly significantly induced in astrocytic mitochondria, which subsequently impairs astrocytic and neuronal function (Park et al., 2021). A previous study demonstrated that nuclear factor erythroid 2-related factor 2 (NRF2, also known as NFE2L2) protects mice from PM_2.5_ exposure-induced depression by inhibiting the NACHT, LRR, and PYD domains containing protein 3 inflammatory pathway using a whole-body NRF2 murine model (Chu et al., 2019). However, the role of NRF2 in specific brain cells, such as astrocytes, in PM_2.5_ exposure-induced depression-like behavior is unclear.

NRF2 transcriptionally regulates enzyme antioxidants, such as glutathione peroxidase, superoxide dismutase (SOD), catalase (CAT), heme oxygenase 1 (HO-1), and peroxidase, in the CNS. It is thus an important transcription factor for maintaining redox balance in the CNS (Cuadrado et al., 2019; Chen et al., 2020). These findings raise concerns about the potential role of astrocytes in the process of PM_2.5_ exposure-induced depression-like behaviors. However, the precise mechanisms and interventions targeting astrocytic polarity and astrocyte-to-microglia communication in real-world PM_2.5_ exposure have not been well investigated.

In the present study, we hypothesized that astrocytic NRF2 may protect mice against PM_2.5_ exposure-induced depression via antioxidation and regulating the communication between astrocytes and microglia. We also aimed to explore whether astrocytic NRF2 is a potential target for interventions against air pollution–induced neural damage.

## Methods

### Particle preparation and characterization

The PM_2.5_ sampling point was located in Tangshan (118°10′26′E longitude, 39°37′46′N latitude), Hebei Province, China. The preparation of PM_2.5_ followed the methodology of a previous report (Guo et al., 2012). PM_2.5_ was characterized using high-resolution transmission electron microscopy (TEM; JEOL, Tokyo, Japan; accelerating voltage of 200 kV) and scanning electron microscopy (JEOL; accelerating voltage of 30 kV).

### PM_2.5_ data sources

Real-time PM_2.5_ data from the location (118°10′26′E longitude, 39°37′46′N latitude) were downloaded from the Tracking Air Pollution (TAP; http://tapdata.org.cn/) database. In the TAP database, data about PM_2.5_ and its components—sulfate (SO_4_^2–^), nitrate (NO_3_^–^), ammonium (NH4^+^), organic matter (OM), and black carbon (BC)—were estimated by combining information from different sources, including ground observations, satellite aerosol optical depth, chemical transport model simulations, meteorological factors, and land-use data. They were calculated on the basis of machine learning, with an out-of-bag cross-validation *R*^2^ of 0.83.

### Hippocampal astrocyte culture

Mice (20–25 g, purchased from GemPharmatech Co., Ltd., Nanjing, China [license No. SYXK (Su) 2023-0086]) were sacrificed by decapitation within 24 hours after birth. Subsequently, the hippocampus was collected under a dissecting (stereo) microscope (Olympus, Tokyo, Japan). Hippocampal tissue was dissociated into single cells, inoculated in a culture flask encapsulated with 0.03% polylysine (Thermo Fisher Scientific, Waltham, MA, USA), and maintained in Dulbecco’s Modified Eagle Medium/F12 medium (Thermo Fisher Scientific) containing 12% fetal bovine serum (Thermo Fisher Scientific) and 1% penicillin/streptomycin (Thermo Fisher Scientific) at 37°C with 5% CO_2_. Purified and cultured astrocytes at 85% confluence were termed the F0 generation. Astrocytes in the F2 generation were used for *in vitro* experiments.

### PM_2.5_ and dimethyl fumarate/phytochemical treatment *in vitro*

Astrocytes were exposed to different doses of PM_2.5_ (0, 5, 10, 20, 40, 80, or 160 μg/mL) for 48 hours. Cell viability was then measured using a Cell Counting Kit-8 (Biosharp, Shanghai, China) following the manufacturer’s instructions.

### Dimethyl fumarate/phytochemical treatment

Dimethyl fumarate (DMF) (Selleck, Shanghai, China) is an NRF2 agonist (Robledinos-Anton et al., 2019). To determine the concentrations of DMF and three phytochemicals (procyanidin [PC; Xi’an Xuhuang Bio-Tech, Xi’an, China], lyceum barb arum polysaccharide [LBP; Xi’an Xuhuang Bio-Tech], and phillyrin [Xi’an Xuhuang Bio-Tech]), astrocytes were exposed to different concentrations (0, 10, 20, 30, 40, 50, 100, 150, or 200 μg/mL) of DMF, PC, LBP, or phillyrin for 2 hours. After treatment, cell viability was measured using a Cell Counting Kit-8 (Biosharp) according to the manufacturer’s instructions.

### Animal and treatments

All animal experiments were approved by the Animal Use and Ethics Committee of Capital Medical University on April 4, 2023 (approval No. AEEI-2023-063) and were conducted in compliance with the National Institutes of Health Guide for the Care and Use of Laboratory Animals (8^th^ ed., National Research Council, 2011). All experimental treatments followed the 3R (replacement, reduction, and refinement) principle. Mice (6–8 weeks, 20–25 g, GemPharmatech Co., Ltd.) were housed in a specific pathogen–free animal facility under a 12-hour dark/light cycle with free access to food and water. The ambient temperature was 22 ± 2°C. The relative humidity of the environment was maintained at 60% ± 10%. Male mice were used for all experiments and were randomly allocated to the experimental and control groups. All mice were maintained on a C57BL/6J background. After 1-week adaptation period, mice were exposed to control (filter room air) or real-world PM_2.5_ for 15 weeks. The real-world PM_2.5_ exposure system was designed according to a previous study (Li et al., 2019). The concentration of ambient PM_2.5_ was measured outdoors and inside individually ventilated cages.

pAAV-GfaABC1D-GFP-miR short hairpin RNA (shRNA) (*Nfe212*)-Woodchuck Response Element (serotype AAV2/5, 8.83 × 10^12^ genome copies/mL) were purchased from OBIO Technology (Shanghai, China). Adult mice were anesthetized using isoflurane (RWD, Shenzhen, China; flow velocity: 300–500 mL/min) before the viruses were stereotaxically injected (flow velocity: 50 nL/min, volume: 500 nL) into the bilateral hippocampus (KOPF brain stereotactic locator, David Koppf Instruments, Tujunga, CA, USA). The mice were stereotaxically injected vectors encoding shRNA targeting *Nfe2l2* (shRNA-Nfe2l2, *Nfe2l2* knockdown [KD] group) or shRNA control (shRNA-NC, NC group) following 15 weeks of PM_2.5_ exposure. The coordinates for stereotaxic injection were as follows (Jiang et al., 2023): anteroposterior –2.1 mm from bregma, mediolateral ±1.5 mm, and dorsoventral –1.5 mm. Mice were then allowed to recover in cages placed partially on a low-voltage heating pad for 2 hours.

The PC was dissolved in phosphate-buffered solution (PBS). The mice were randomly divided into four groups (Control, PC (100 mg/kg/d), PM_2.5_, and PC + PM_2.5_). The test period was 15 weeks.

### Quantitative reverse transcription-polymerase chain reaction analysis

After 15 weeks of PM_2.5_ exposure and behavioral testing, mice were anesthetized using 4% isoflurane, and their brains were swiftly extracted and placed in ice-cold PBS. Total RNA of primary cultured astrocytes and the murine hippocampus was extracted using an RNA isolation reagent (Thermo Fisher Scientific) following the manufacturer’s instructions. Complimentary DNA was then transcribed from 2 μg of RNA using a TaqMan MiRNA Reverse Transcription Kit (Takara, Osaka, Japan). The reverse transcription program was set at 37°C for 15 minutes, 85°C for 5 seconds, and 4°C for holding. The quantitative reverse transcription-polymerase chain reaction analysis was performed using a SYBR Premix Ex Taq kit (Takara) using the Quant Studio 6 Flex system (Applied Biosystems, Hercules, CA, USA). β-Actin was used to quantify the relative expression of target genes using the 2–ΔΔCt method. The reaction conditions included an initial step of 30 seconds at 95°C, which was then followed by 40 cycles of 2–10 seconds at 95°C and 10–30 seconds at 60°C, with a final step of 60 seconds at 60°C and 15 seconds at 95°C. The primer information is shown in **Additional Table 1**.

**Additional Table 1:** The primer information for quantitative reverse transcription-polymerase chain reaction assays

Gene	Primer (5'-3')
	F: GGCTGTATTCCCCTCCATCG-
*β-actin*	R: CCAGTTGGTAACAATGCCATGT-
	F: TGGTTGCCCTCATTGATGTCT-
*S100-β*	R: CCCATCCCCATCTTCGTCC-
	F: CCCTGGCTCGTGTGGATTT-
*Gfap*	R: GACCGATACCACTCCTCTGTC-
	F: CCAGCTCCCCATTAGCTCTG-
*C3*	R: GCACTTGCCTCTTTAGGAAGTC-
	F: TCTTGGAGTAAGTCGAGAAGTGT-
*Nrf2*	R: GTTGAAACTGAGCGAAAAAGGC-
	F: AAGCCGAGAATGCTGAGTTCA-
*Ho-1*	GCCGTGTAGATATGGTACAAGGA
	F: ATGGGAGGTGGTCGAATCTGA-
*Nqo-1*	R: GCCTTCCTTATACGCCAGAGATG-
	F: CAGGGCCTGATGATGAAGCG
*Relt*	R: GGGGCAAGTTCTGCATAATGTG
	F: GCAGGATGTACTCCCCTGG
*Mafg*	R: CCAAGCCCTAGACCCACAATG
	F: TTGTGGTATTACGCCTGTGTATC
*Jak2*	R: ATGCCTGGTTGACTCGTCTAT
	F: GCTGAGAGCATCCGACTGAAC
*Nlrc5*	R: AGGTACATCAAGCTCGAAGCA
	F: CCAAGTGCTGCCGTCATTTTC
*Cxcl10*	R: GGCTCGCAGGGATGATTTCAA
	F: TACCTTGAGGTTAGTGAACGTCA
*Cxcr3*	R: CGCTCTCGTTTTCCCCATAATC
	F: CAATACCATTGACCTGCCGAT
*Stat3*	R: GAGCGACTCAAACTGCCCT
	F: ATGGCAGACGATGATCCCTAC
*NF-κB*	R: TGTTGACAGTGGTATTTCTGGTG
	F: AGAGGGGAATGCGGTTTAGAT
*Rel*	R: TTCTGGTCCAAATTCTGCTTCAT
	F: TTAAAAACCTGGATCGGAACCAA
*Ccl2*	R: GCATTAGCTTCAGATTTACGGGT

### Western blot analysis

After 15 weeks of PM_2.5_ exposure and behavioral testing, mice were anesthetized using 4% isoflurane (RWD), and their brains were swiftly extracted and placed in ice-cold PBS. Total proteins were extracted from primary cultured astrocytes and the murine hippocampus using a total protein extraction kit (Thermo Fisher Scientific). A total of 15 μg of protein from each sample underwent 8% or 10% sodium dodecyl sulfate-polyacrylamide gel electrophoresis (Yazyme Biotech, Shanghai, China). Separated proteins were then transferred onto polyvinylidene difluoride membranes (Millipore, Burlington, MA, USA). Next, the membranes were blocked with 5% bovine serum albumin (BSA; Solarbio, Beijing, China) for 1 hour before being incubated for 12 hours with the following primary antibodies: rabbit anti-complement component 3 (C3; 1: 500, Cat# Ab97462, Abcam, Cambridge, UK), rabbit anti-Nrf2 (1: 200, Cat# 12721S, Cell Signaling Technology, Danvers, MA, USA), rabbit anti-HO-1 (1:1000, Cat# 10701-1-AP, Proteintech, Wuhan, China), rabbit anti-NAD(P)H dehydrogenase [quinone] 1 (NQO-1; 1:1000, Cat# 11451-1-AP, Proteintech), and rabbit anti-β-actin (1:5000, Cat# 8226, Abcam). This was followed by incubation with horseradish peroxidase-conjugated secondary antibodies for 1 hour (anti-rabbit IgG: 1:3000, Cat# 7074, Cell Signaling Technology). Images were developed using a Super ECL Plus Kit (Yeasen, Shanghai, China). The analysis of corresponding gray values in images was conducted using ImageJ (v1.8.0, National Institutes of Health, Bethesda, MD, USA). Relative protein expression was normalized to β-actin.

### Histopathological and immunohistochemical staining analysis

After 15 weeks of PM_2.5_ exposure and behavioral testing, mice were anesthetized using 4% isoflurane (RWD), and their brains were swiftly extracted and placed in ice-cold PBS. Mouse brain tissue was collected and fixed with 4% paraformaldehyde. After dehydration, samples were embedded in paraffin and cut into 3-μm sections. The sections were dewaxed through a gradient before being stained with hematoxylin and eosin (Beyotime, Shanghai, China). For the immunohistochemical staining, microwave antigen retrieval was performed using citric acid buffer (pH 6.0), and tissue sections were cooled in Tris-buffered saline-Tween 20 containing 5% BSA for 30 minutes at room temperature. Next, the sections were incubated for 24 hours at 4°C with the following primary antibodies: mouse anti-GFAP (1:250, Cat# 3670, Cell Signaling Technology), rabbit anti-C3 (1:500, Cat# Ab97462, Abcam), rabbit anti-ionized calcium binding adaptor molecule 1 (Iba1; 1:600, Cat# ab17884, Abcam). Thereafter, the sections were incubated in the corresponding biotinylated secondary antibodies using a Universal Two-Step Test Kit (Nakasugi, Beijing, China) in blocking solution (3%) for 1 hour at room temperature and developed with 3,3-diaminobenzidine (Nakasugi). Images were then scanned using a Zeiss Laser Scanning Microscope 710 (Carl Zeiss, Baden-Wurttemberg, Germany). Non-overlapping high-power fields (400) were captured using a Panoramic SCAN (3DHISTECH, Budapest, Hungary). Immunostaining intensity or the number of GFAP^+^ cells in each field was evaluated.

### Immunofluorescence staining

After 15 weeks of PM_2.5_ exposure and behavioral testing, mice were anesthetized using 4% isoflurane (RWD), and their brains were swiftly extracted and placed in ice-cold PBS. After dewaxing and antigen retrieval, sections of brain tissue were incubated with 5% BSA at room temperature for 30 minutes. In addition, astrocytes were fixed with 4% paraformaldehyde, permeabilized, and sealed with 5% BSA containing 0.3% Triton X-100 (Solarbio) for 1 hour. After being washed, the samples were incubated with the following primary antibodies: mouse anti-GFAP (1:250, Cat# 3670, Cell Signaling Technology), rabbit anti-C3 (1:500, Cat# Ab97462, Abcam), rabbit anti-Nrf2 (1:200, Cat# 12721S, Cell Signaling Technology), and rabbit anti-CD68 (1:100, Cat# sc-20060, Santa Cruz Biotechnology). This was followed by incubation with goat anti-mouse IgG secondary antibody conjugated with Alexa Fluor 488 (1:400, Cat# A-11001, Thermo Fisher Scientific) and goat anti-rabbit IgG secondary antibody conjugated with Alexa Fluor 555 (1: 400, Cat# A-21424, Thermo Fisher Scientific). After the samples were stained with 4′,6-diamidino-2-phenylindole, images were taken using a confocal microscopy (Carl Zeiss). Average fluorescence intensity was measured using ImageJ, and protein expression was semi-quantitatively analyzed.

### Flow cytometry assay

After 48 hours of PM_2.5_ exposure, primary cultured astrocytes were collected and probed with a reactive oxygen species (ROS) Assay Kit (Beyotime) according to the manufacturer’s instructions. The numbers of ROS^+^ astrocytes were detected using flow cytometry (BD Life Sciences, Franklin Lakes, NJ, USA).

### Measurement of antioxidant indexes

Astrocytes were collected and lysed in radioimmunoprecipitation assay lysis solution (Thermo Fisher Scientific). The levels of SOD and CAT in astrocytes were measured using a SOD Assay Kit (Beyotime) and a CAT Assay Kit (Beyotime), respectively, following the manufacturer’s instructions.

### Enzyme-linked immunosorbent assay

After 15 weeks of PM_2.5_ exposure and behavioral testing, mice were anesthetized using 4% isoflurane (RWD). Next, peripheral blood serum was collected from the mice, and S100β protein levels were measured using a mouse S100β enzyme-linked immunosorbent assay kit (Beyotime) according to the manufacturer’s instructions.

### Open field test

After 15 weeks of PM_2.5_ exposure, to observe whether mice showed alterations in locomotor activity and anxiety-like behaviors (Kraeuter et al., 2019; Liu et al., 2025), the open field test (OFT) was performed in a 42 × 42 × 42 cm^3^ polyvinyl chloride box. Mouse movements were recorded using a camera (Litong, Deyang, China). Each mouse was tested for 3 minutes. The videos were analyzed using EthpVision XT 17.0 (Noldus, Wageningen, the Netherlands).

### Sucrose preference test

After 15 weeks of PM_2.5_ exposure and the OFT, we used the sucrose preference test (SPT) to observe whether the mice developed depression-like behaviors (Cao et al., 2013). In this test, we measured the sucrose preferences of mice using 1% sucrose solution. The amount of pure water (*M*_water_) and sucrose solution (*M*_sucrose solution_) consumed by mice within 24 hours was measured. The equation for sucrose preference was as follows: sucrose preference (%) = *M*_sucrose solution_ /(*M*_water_ + *M*_sucrose solution_) × 100.

### RNA sequencing analysis

After mice were bilaterally injected with virus followed by 15 weeks of PM_2.5_ exposure and behavioral testing, the mice were anesthetized using 4% isoflurane (RWD) and their brains were swiftly extracted and placed in ice-cold PBS. Total RNA was extracted from the mouse hippocampus using TRIzol (Thermo Fisher Scientific). Transcriptome sequencing experiments were delegated to Novogene Bioinformatics (Beijing, China). The basic principle of sequencing depended on the sequencing by synthesis method. In the data analysis phase, differentially expressed genes (DEGs) with a cut-off of adjusted *P*-value < 0.05 and fold change > 1.5 were identified for further analysis. Kyoto Encyclopedia of Genes and Genomes (KEGG) analyses were performed using R (4.0.2) (de Hoyos et al., 2024). DEGs were subjected to KEGG pathway enrichment analysis using the clusterProfiler package (v4.0) (Xu et al., 2024). Gene identifiers were mapped to KEGG Orthology entries via the KEGG REST API (database version: 2023.01). Enrichment significance was calculated using a hypergeometric test with Benjamini–Hochberg correction (false discovery rate < 0.05). The relative expression of chemokine genes was extracted from RNA sequencing data, and correlation analysis was conducted using the Stat package in R (4.0.2) (de Hoyos et al., 2024).

### Nissl staining

After 15 weeks of PM_2.5_ exposure and behavioral testing, mice were anesthetized using 4% isoflurane (RWD), and their brains were swiftly extracted and fixed in 4% paraformaldehyde before being embedded in paraffin. After dewaxing, the brain tissue was stained for 10 minutes using a Nissl staining kit (Beyotime) according to the manufacturer’s instructions.

### Statistical analysis

No statistical methods were used to predetermine sample sizes; however, our sample sizes were similar to those reported in previous publications (Fan et al., 2025). Data analysis was performed using GraphPad Prism 8.0 software (GraphPad Software, Boston, MA, USA, www.graphpad.com) and the results are presented as the mean ± standard error of the mean. The normal distribution of data within each group was evaluated before the appropriate statistics were performed. Comparisons between two experimental groups were assessed using unpaired two-tailed unpaired *t*-tests or nonparametric Mann‒Whitney *U* tests. Differences among multiple groups were measured using one-way analysis of variance followed by Tukey’s multiple comparisons test and two-way analysis of variance followed by Dunnett’s multiple comparisons test. *P* < 0.05 was set as the significance level.

## Results

### Long-term real-world PM_2.5_ exposure leads to depression-like behaviors in mice

**Figure 1:**
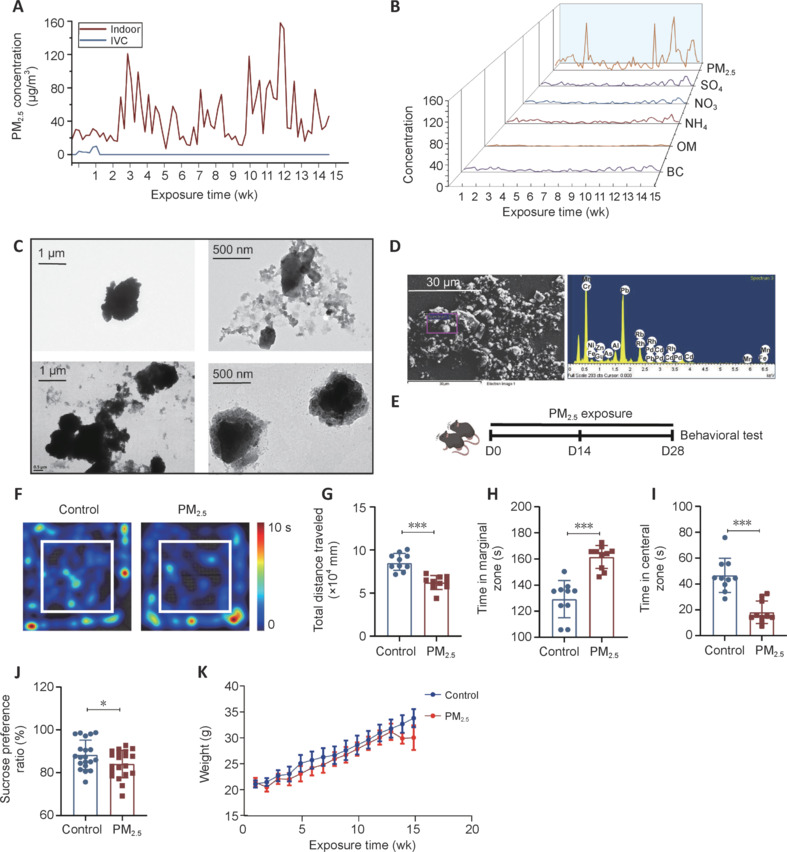
Long-term real-world fine particulate matter (PM_2.5_) exposure leads to depression-like behaviors in male mice. (A) Daily concentrations of PM_2.5_ indoor and in individually ventilated cages (IVC) during a 15-week exposure period to PM_2.5_. (B) Daily concentrations of ambient PM_2.5_ and its components from the Tracking Air Pollution (TAP) database: sulfate (SO_4_^2–^), nitrate (NO_3_^–^), ammonium (NH4^+^), organic matter (OM), and black carbon (BC). (C) Transmission electron microscopy (TEM) images of PM_2.5_. (D) High-resolution scanning electron microscope (HRSEM) mapping of PM_2.5_. (E) Schematic illustration of real-world PM_2.5_ exposure and behavioral and histological assessments in mice. (F) Representative heatmaps indicating the movements of control and PM_2.5_-exposed mice in the open field test (OFT). (G–I) Total distance moved (*t* = 5.890, *df* = 18, *P* < 0.0001, *F* = 1.483, *n* = 10) and time spent in the marginal (*t* = 6.077, *df* = 18, *P* < 0.0001, *F* = 2.659, *n* = 10) and central zones (*t* = 5.711, df = 18, *P* < 0.0001, *F* = 2.335, *n* = 10) of control and PM_2.5_-exposed mice in the OFT (*n* = 10 biological replicates/group, unpaired *t*-test). (J) Sucrose consumption (*t* = 2.686, *df* = 18, *P* = 0.0151, *F* = 1.060, *n* = 10) of control and PM_2.5_-exposed mice in the sucrose preference test (SPT; *n* = 10 biological replicates/group, unpaired *t*-test). (K) Weight of control and PM_2.5_-exposed mice (*n* = 20 biological replicates/group). D: Day; IVC: individually ventilated cage; PM2.5: ambient fine particulate matter.

To explore the effects of PM_2.5_ on depressive-like behaviors, mice were continuously exposed to ambient PM_2.5_. During the exposure period, the average concentration of PM_2.5_ inside the individually ventilated cage was 49.76 ± 32.64 μg/m^3^ (**Figure 1A**), which was detected using an air particulates measurement meter. The main components in ambient PM_2.5_ were as follows: SO_4_^2−^: 1.69 ± 1.11 μg/m^3^, NO_3_^–^: 6.05 ± 4.97 μg/m^3^, NH4^+^: 8.46 ± 8.14 μg/m^3^, OM: 5.31 ± 4.38 μg/m^3^, and BC: 8.38 ± 5.5 μg/m^3^ (**Figure 1B**). These values were obtained from the TAP database. Representative TEM images and analyses revealed that the size distribution of PM_2.5_ in the exposure chamber mainly ranged from 0.2–1 μm (**Figure 1C**). The scanning electron microscopy revealed that the PM_2.5_ particles contained small metal impurities, which were mainly Mn, Cr, and Pb, with very little Fe, Rb, Rh, Cd, Ni, Zn, Al, Gu, and As (**Figure 1D**).

To clarify the direct effects of real-world PM_2.5_ exposure on neural behaviors, mice were continuously exposed to filter room air (control group) or ambient PM_2.5_ (PM_2.5_ group) for 15 weeks using a whole-body exposure system (**Figure 1E**). Following exposure, we assessed the depression-like behaviors of mice using the OFT and SPT. Compared with the control, PM_2.5_-exposed mice exhibited a significantly shorter total moving distance (*P* < 0.0001), spent less time in the central area (*P* < 0.0001) of the OFT (**Figure 1F–H**), and spent a considerably longer time in the marginal zone (*P* < 0.0001) (**Figure 1I**), indicating decreased locomotor activity in PM_2.5_-exposed mice. In addition, the SPT revealed that the sucrose preference rate of mice in the PM_2.5_ group was significantly lower (*P* = 0.0151) than that in the control group (**Figure 1J**). However, there was no noticeable difference in body weight between the control and PM_2.5_-exposed groups (**Figure 1K**). Collectively, our results indicate that long-term real-world PM_2.5_ exposure leads to depressive-like behaviors in mice.

### Ambient PM_2.5_ exposure induces pathological damage in murine hippocampal neurons

**Figure 2:**
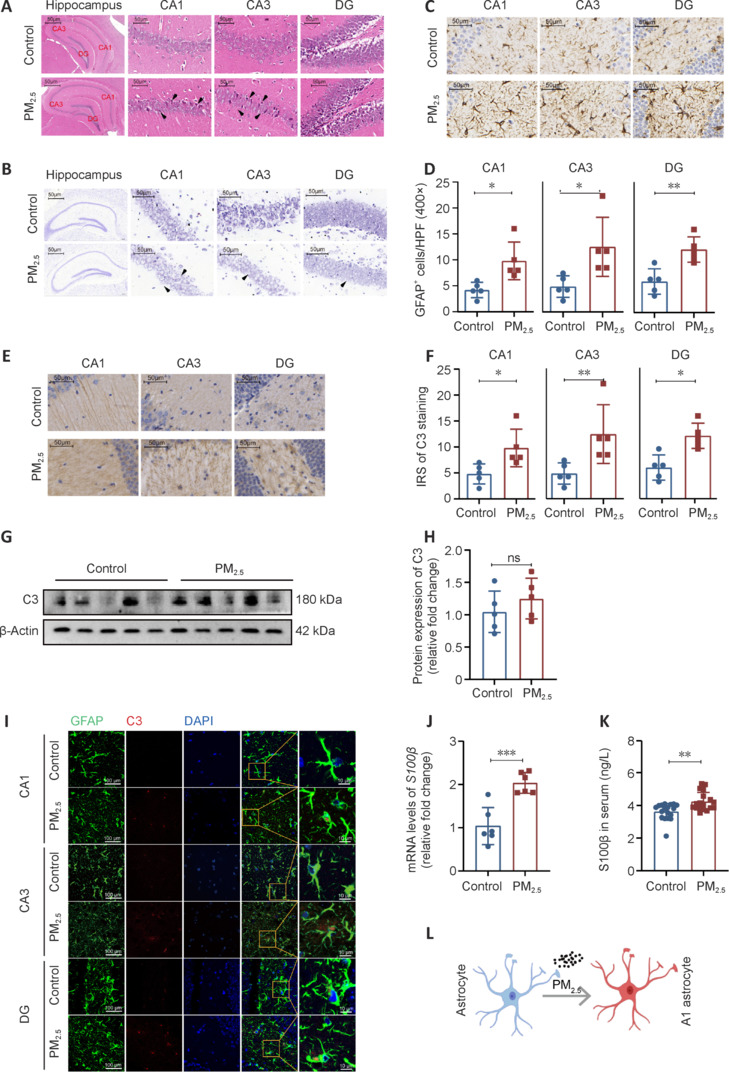
Chronic fine particulate matter (PM_2.5_) exposure induces pathological damage in mouse neurons through the induction of hippocampal astrocytic reactivity. (A, B) Representative images of hematoxylin and eosin (A) or Nissl (B) staining in brain sections of control and PM_2.5_-exposed mice. Scale bars: 50 μm. (C, D) Representative images of anti-glial fibrillary acidic protein (GFAP) staining in mouse brain sections (C) and the numbers of GFAP-positive cells per high-power field (400×) (D; cornu ammonis 1 [CA1]: *t* = 3.191, *df* = 8, *P* = 0.0128, *F* = 6.000, *n* = 5, CA3: *t* = 2.824, *df* = 8, *P* < 0.0224, *F* = 7.553, *n* = 5, dentate gyrus [DG]: *t* = 3.983, *df* = 8, *P* = 0.0040, *F* = 1.000, *n* = 5) of control and PM_2.5_-exposed mice. Scale bars: 50 μm. (*n* = 5 biological replicates/group, unpaired *t*-test). (E, F) Representative images (E) and relative quantification (F) of anti-complement component 3 (C3) staining in mouse brain sections, and the immunoreactive score (IRS) of C3 staining (CA1: *P* = 0.0159, *n* = 5, CA3: *P* = 0.0079, *n* = 5, DG: *P* = 0.159, *n* = 5) of control and PM_2.5_-exposed mice (*n* = 5 biological replicates/group, Mann–Whitney *U* test). Scale bars: 50 μm. (G, H) Relative expression of C3 (*t* = 1.018, d*f* = 8, *P* = 0.3384, *F* = 1.038, *n* = 5) in control and PM_2.5_-exposed mice using western blot analysis (*n* = 5 biological replicates/group, unpaired *t*-test). (I) Co-expression of GFAP and C3 in the CA1, CA3, and DG regions of control and PM_2.5_-exposed mice. Fields of view were captured at 100× magnification. (J) Relative mRNA expression of *S100*β using quantitative reverse transcription polymerase chain reaction (*t* = 4.968, *df* = 10, *P* = 0.0006, *F* = 3.150, *n* = 6) in control and PM_2.5_-exposed mice (*n* = 7 biological replicates/group, unpaired *t*-test). (K) Enzyme-linked immunosorbent assay was used to detect the levels of S100 calcium-binding protein B (S100β; *t* = 3.091, *df* = 44, *P* = 0.0035, *F* = 1.025, *n* = 20) in control and PM_2.5_-exposed mice (*n* = 20/group, biological replicates, unpaired *t-*test). *P < 0.05, **P < 0.01, ***P < 0.01. (L) Schematic illustration of astrocytic toxicity induced by PM2.5 Exposure. GFAP: Glial fibrillary acidic protein; IRS: immune response score; PM_2.5_: ambient fine particulate matter; S100β: S100 calcium-binding protein B.

The hippocampus is a brain region that is associated with emotion regulation, and the disruption of hippocampal function plays a vital role in depression occurrence (Du Preez et al., 2021; Somelar et al., 2021). In untreated mice, hippocampal neurons in the cornu ammonis 1 (CA1), CA3, and dentate gyrus (DG) were arranged in an orderly and compact manner, with distinct nucleoli and uniform cytoplasm. However, neurons in the hippocampal regions of PM_2.5_-exposed mice exhibited obscure cell layers with a disorderly arrangement and irregular morphology; some neurons were darkly stained and cell bodies were shrunken, indicating the severity of hippocampal injury in PM_2.5_-exposed mice (**Figure 2A**). Nissl staining was used to detect further hippocampal alterations in mice with PM_2.5_-induced depression-like behaviors (**Figure 2B**). Following PM_2.5_ exposure, Nissl bodies in CA1, CA3, and DG neurons were reduced and showed unclear stratification and a loose arrangement compared with the control group. These results provide further evidence to suggest that long-term ambient PM_2.5_ exposure in mice induces neuronal damage in the hippocampus during the pathogenesis of depressive-like behaviors.

### PM_2.5_ exposure promotes the polarization of astrocytes to an A1 phenotype in the murine hippocampus

Anti-GFAP staining revealed the activation of murine astrocytes in the CA1, CA3, and DG after PM_2.5_ exposure, which was observed as enlarged cell bodies, thick processes, and dark staining (**Figure 2C**). Furthermore, the number of GFAP^+^ cells was significantly higher (CA1: *P* = 0.0128, CA3: *P* < 0.0224, DG: *P* = 0.0040) in the PM_2.5_-exposed group than in the control group (**Figure 2D**). By contrast, Iba1^+^ microglia had no apparent differences in number and morphology between the two groups (**Additional Figure 1**). These findings indicate that ambient PM_2.5_ exposure facilitates the transformation of normal astrocytes into reactive astrocytes with A1 subtypes (Anderson et al., 2016). Given that A1 astrocytes contribute to the death of neurons and are linked to depressive behaviors (Zhang et al., 2020), we performed immunohistochemistry and western blot analysis on murine brain tissue to assess the expression of C3 (a marker of A1 astrocytes) (Liddelow et al., 2017). C3 protein expression in the CA1, CA3, and DG was higher (CA1: *P* = 0.0159, CA3: *P* = 0.0079, DG: *P* = 0.0159) in the PM_2.5_-exposed group than in the control group (**Figure 2E** and **F**). Moreover, the expression levels of C3 protein in the brain were slightly higher (*P* = 0.3384) in the PM_2.5_ group than in the control group (**Figure 2G** and **H**).

Increased GFAP content is a strong indicator of astrocyte remodeling, and astrocytic polarization is associated with the occurrence and progression of depression (Escartin et al., 2021). Compared with the control group, the fluorescence intensity of GFAP and C3 protein was increased in the PM_2.5_-exposed group, and GFAP^+^/C3^+^ cells in the CA1, CA3, and DG were enhanced, indicating that PM_2.5_ exposure triggers A1 activation in astrocytes (**Figure 2I**). S100β is a commonly used marker for diagnosing neurological disorders such as neuroinflammation, depression, neurodegeneration, and brain injury (Du Preez et al., 2021; Langeh and Singh, 2021). S100β is secreted from astrocytes, and astrocytic activation reduces hippocampal neurogenesis in chronic stress-induced depressive murine models (Du Preez et al., 2021). In the present study, S100β levels were significantly higher (*S100b* mRNA: *P* = 0.0006, S100β protein: *P* = 0.0035) in the brain tissue and serum of PM_2.5_-exposed mice relative to control mice (**Figure 2J** and **K**). Together, our results suggest that long-term real-world PM_2.5_ exposure promotes the polarization of astrocytes to the A1 phenotype in the CA1, CA3, and DG of the murine hippocampus (**Figure 2L**).

### Inhibition of Nrf2 activation is a potential mechanism of astrocytic A1 polarization in the hippocampus after chronic real-world PM_2.5_ exposure

**Figure 3:**
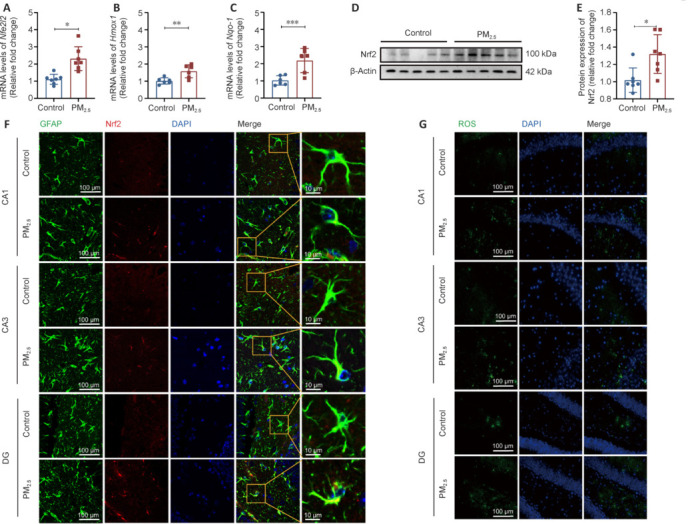
Inhibition of nuclear factor erythroid 2-related factor 2 (Nrf2) activation is a potential mechanism of astrocytic reactivity in the hippocampus after real-world fine particulate matter (PM_2.5_) exposure. (A–C) Relative expression of *Nfe2l2* (*t* = 2.715, *df* = 14, *P* = 0.0168, *F* = 17.57, *n* = 8), *Hmox1* (*t* = 3.411, *df* = 14, *P* = 0.0042, *F* = 4.187, *n* = 8), and *Nqo-1* (*t* = 4.146, *df* = 14, *P* = 0.0010, *F* = 7.383, *n* = 8) using quantitative reverse transcription polymerase chain reaction in control and PM_2.5_-exposed mice (*n* = 6/7/group, unpaired *t*-test). (D, E) Relative expression of Nrf2 (*t* = 4.111, *df* = 8, *P* = 0.0034, *F* = 1.584, *n* = 5) using western blot analysis in control and PM_2.5_-exposed mice (*n* = 5, unpaired *t*-test). *P < 0.05, **P < 0.01, ***P < 0.01. (F) Co-expression of glial fibrillary acidic protein (GFAP) and Nrf2 in the cornu ammonis (CA)1, CA3, and dentate gyrus (DG) regions of control and PM_2.5_-exposed mice. Fields of view were captured at 100× magnification. (G) ROS generation in the CA1, CA3, and DG regions of control and PM_2.5_-exposed mice. Fields of view were captured at 100× magnification. DG: Dentate gyrus; DAPI: 4′,6-Diamidino-2-phenylindole; GFAP: glial fibrillary acidic protein; PM_2.5_: ambient fine particulate matter.

To elucidate the possible mechanism underlying long-term real-world PM_2.5_ exposure-driven astrocytic reactivity in the murine hippocampus, we analyzed the key genes involved in astrocytic polarization-related signaling following PM_2.5_ exposure (**Figure 3A–C** and **Additional Figure 2**) and the expression of pathway genes, as previously reported (Colombo and Farina, 2016). Nrf2 is a regulator of antioxidant signaling during neuroinflammation, and its dysregulation is involved in the neuronal injuries that are observed in depression (Zhang et al., 2023). Compared with control mice, PM_2.5_ exposure led to significantly higher hippocampal mRNA expression of Nrf2-related genes, including *Nfe2l2* (*P* = 0.0168), *Hmox1* (*P* = 0.0042), and *Nqo-1* (*P* = 0.0010) (**Figure 3A–C**); these findings were similar to the level of hippocampal Nrf2 protein in PM_2.5_-exposed mice as determined by western blot analysis (**Figure 3D** and **E**), indicating that Nrf2 signaling in the hippocampus is activated after real-world PM_2.5_ exposure. On the basis of our immunofluorescence results, we further confirmed that Nrf2 protein level, ROS generation, and GFAP^+^/Nrf2^+^ cells increased markedly in CA1, CA3, and DG hippocampal astrocytes (**Figure 3F** and **G**).

To further explore whether NRF2 activation in astrocytes following PM_2.5_ exposure is relevant to the initiation of neuronal injury, primary astrocytes were obtained from newborn mice (postnatal day 0.5) and cultured for 10 days to evaluate NRF2 activation and astrocytic A1 polarization *in vitro* following PM_2.5_ exposure (**Additional Figure 3A**). Compared with the untreated astrocytes, PM_2.5_-exposed primary astrocytes exhibited lower cell viability (**Additional Figure 3B**), higher mRNA and protein expression levels of GFAP and C3 (**Additional Figure 3C–F**), and more GFAP^+^/C3^+^ cells (**Additional Figure 3G**), indicating that astrocytic A1 polarization occurs following PM_2.5_ exposure. Exposure by primary astrocytes significantly increased the expression of *Nfe2l2* (0 μg/mL *vs*. 5 μg/mL: *P* = 0.011, 0 μg/mL *vs.* 10 μg/mL: *P* = 0.0006, 0 μg/mL *vs.* 15 μg/mL: *P* < 0.0001), *Hmox1* (0 μg/mL *vs.* 5 μg/mL: *P* = 0.0015, 0 μg/mL *vs.* 10 μg/mL: *P* = 0.0002, 0 μg/mL *vs.* 15 μg/mL: *P* < 0.0001), and *Nqo-1* (0 μg/mL *vs*. 5 μg/mL: *P* = 0.0120, 0 μg/mL *vs*. 10 μg/mL: *P* = 0.0001, 0 μg/mL *vs.* 15 μg/mL: *P* < 0.0001) mRNA (**Additional Figure 3H–J**), and of NRF2 (0 μg/mL *vs.* 5 μg/mL: *P* = 0.0871, 0 μg/mL *vs.* 10 μg/mL: *P* = 0.0094, 0 μg/mL *vs*. 15 μg/mL: *P* = 0.0002), HO-1 (0 μg/mL *vs*. 5 μg/mL: *P* = 0.1867, 0 μg/mL *vs.* 10 μg/mL: *P* = 0.0067, 0 μg/mL *vs.* 15 μg/mL: *P* = 0.0019), and NQO-1 (0 μg/mL *vs.* 5 μg/mL: *P* = 0.2123, 0 μg/mL *vs*. 10 μg/mL: *P* = 0.0440, 0 μg/mL *vs*. 15 μg/mL: *P* = 0.0109) protein (**Additional Figure 3K–N**) compared with the control cells. Furthermore, GFAP^+^/C3^+^ cells were significantly enhanced in PM_2.5_-exposed primary astrocytes (**Additional Figure 3O**). PM_2.5_ exposure also disturbed the redox balance in astrocytes, with significantly higher CAT (0 μg/mL *vs*. 5 μg/mL: *P* = 0.1052, 0 μg/mL *vs.* 10 μg/mL: *P* = 0.0096, 0 μg/mL *vs.* 15 μg/mL: *P* = 0.0032), SOD capacities (0 μg/mL *vs.* 5 μg/mL: *P* = 0.4678, 0 μg/mL *vs.* 10 μg/mL: *P* = 0.0271, 0 μg/mL *vs.* 15 μg/mL: *P* = 0.0035), and ROS generation (0 μg/mL *vs.* 5 μg/mL: *P* = 0.1163, 0 μg/mL *vs.* 10 μg/mL: *P* = 0.0345, 0 μg/mL *vs*. 15 μg/mL: *P* = 0.0002) in PM_2.5_-exposed primary astrocytes than in control astrocytes (**Additional Figure 3P–S**). These results further suggest that NRF2 signaling is critical for astrocytic A1 polarization.

### Downregulation of hippocampal nuclear factor erythroid 2-related factor 2 in astrocytes aggravates PM_2.5_-induced depressive-like behaviors by dampening astrocyte-to-microglia communication

**Figure 4:**
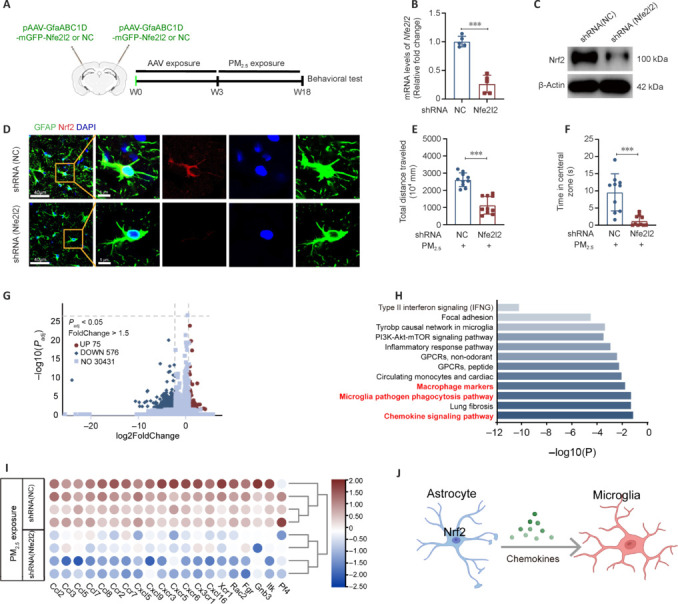
Bilateral downregulation of hippocampal nuclear factor erythroid 2-related factor 2 (NRF2) in astrocytes aggravates fine particulate matter (PM_2.5_)-induced depression-like behaviors by dampening astrocyte-to-microglia communication. (A) Schematic of the construction of a bilateral astrocytic *Nfe2l2* knockdown (KD) mouse model by the bilateral hippocampal injection of adenoviral vectors encoding short hairpin RNA (shRNA) targeting the gene encoding NRF2 (shRNA-*Nfe2l2*, *Nfe2l2* KD group) or control shRNA (shRNA-NC, NC group), followed by 15 weeks of PM_2.5_ exposure. (B, C) mRNA and protein levels of Nrf2 (*t* = 12.61, *df* = 6, *P* < 0.0001, *F* = 10.71, *n* = 4) in the hippocampus of mice in the *Nfe2l2* KD and NC groups after 15 weeks of PM_2.5_ exposure (*n* = 5 biological replicates/group). (D) Co-expression of glial fibrillary acidic protein (GFAP) and Nrf2 in *Nfe2l2* KD and NC astrocytes in the murine brain. Fields of view were captured at 100× magnification. (E, F) Total distance moved (*t* = 7.163, *df* = 18, *P* < 0.0001, *F* = 1.670, *n* = 10) and time spent in the marginal (*t* = 1.408, *df* = 18, *P* = 0.1716, *F* = 3.675, *n* = 10) and central (*t* = 4.703, *df* = 6, *P* = 0.0002, *F* = 12.70, *n* = 10) zones of *Nfe2l2* KD and NC mice after 15 weeks of PM_2.5_ exposure, in the open field test (OFT; *n* = 10/group, biological replicates). **P < 0.01, ***P < 0.01 (unpaired *t*-test). (G) Volcano plot of RNA sequencing data from the hippocampus of *Nfe2l2* KD and NC group mice after 15 weeks of PM_2.5_ exposure (*n* = 4/group). Red dots: Upregulated differentially expressed genes (DEGs); blue dots: downregulated DEGs (*n* = 4/group). *F*-tests were used for statistical testing. The cut-off was set as *P* < 0.05 and fold change > 1.5. (H) Kyoto Encyclopedia of Genes and Genomes (KEGG) enrichment analysis for downregulated DEGs relative to those in Nfe2l2 NC brain tissue. (I) Heatmap of downregulated DEGs in the chemokine signaling pathway. (J) Schematic illustrating that astrocytic NRF2 KD inhibits connections between astrocytes and microglia by decreasing cytokine levels. DAPI: 4′,6-Diamidino-2-phenylindole; GFAP: glial fibrillary acidic protein; KD: knockdown; KEGG: Kyoto Encyclopedia of Genes and Genomes; NC: normal control; PM_2.5_: ambient fine particulate matter; W: week.

To explore the role of astrocytic NRF2 in PM_2.5_-induced depressive-like behaviors in mice, astrocytic NRF2 expression in mice was inhibited by the bilateral hippocampal injection of adenoviral vectors encoding shRNA targeting *Nfe2l2* (shRNA-Nfe2l2, Nfe2l2 KD group) or shRNA control (shRNA-NC, NC group) following 15 weeks of PM_2.5_ exposure (**Figure 4A** and **Additional Figure 4B**). The mRNA and protein levels of NRF2 were reduced by approximately 70% in mice that received *Nfe2l2* KD (**Figure 4B** and **C**). Furthermore, NRF2 expression was ablated in GFAP^+^ astrocytes (**Figure 4D**). During exposure, the average concentration of PM_2.5_ was 36.05 ± 21.42 μg/m^3^ and its components were 1.41 ± 0.84 μg/m^3^ (BC), 8.22 ± 5.60 μg/m^3^ (OM), 5.44 ± 3.55 μg/m^3^ (NH4), 8.41 ± 5.98 μg/m^3^ (NO_3_), and 6.73 ± 4.79 μg/m^3^ (SO_4_) (**Additional Figure 4A**).

Next, we sought to determine the *in vivo* functional consequences of astrocyte-specific NRF2 loss on PM_2.5_-induced depressive-like behaviors. Consistent with more severe depressive-like behaviors, PM_2.5_-exposed *Nfe2l2* KD mice exhibited a shorter total moving distance and spent less time in the central area of the OFT compared with NC mice (**Figure 4E** and **F**). Thus, the astrocytic ablation of NRF2 induced hippocampal neuronal injury and exacerbated the development of PM_2.5_-induced depressive-like behaviors in mice.

Subsequently, we investigated the hippocampal mRNA expression profiles via RNA sequencing analysis to elucidate the possible mechanisms that regulate depressive-like behaviors in NC and *Nfe2l2* KD mice following 15 weeks of PM_2.5_ exposure. As shown in the volcano plot (**Figure 4G**), 651 DEGs were identified (adjusted *P*-value < 0.05, fold change > 1.5), of which 576 genes were downregulated and 75 genes were upregulated in the hippocampus of *Nfe2l2* KD mice compared with NC mice. KEGG analysis of these DEGs revealed that the downregulated genes were predominantly involved in astrocyte-to-microglia communication, including chemokine signaling, microglia pathogen phagocytosis, macrophage markers, and type II interferon signaling (**Figure 4H**).

Disruption of astrocyte-to-microglia communication plays a critical role in the pathophysiological phenotypes of depression, and chemokines produced by astrocytes have been identified as a crucial mediator of astrocyte–microglia crosstalk in nervous system diseases (Takei et al., 2023). We also performed a heatmap analysis of the downregulated DGEs and noted that a loss of hippocampal NRF2 in astrocytes significantly decreased PM_2.5_-induced astrocytic chemokine signaling (**Figure 4I** and **Additional Figure 4C–H**), including of *Cxcl* and *Ccl* family numbers. Collectively, these findings suggest that astrocytic *Nfe2l2* KD disrupts chemokine signaling-mediated microglial function (**Figure 4J**) and aggravates depressive-like behaviors in PM_2.5_-exposed mice.

**Figure 5:**
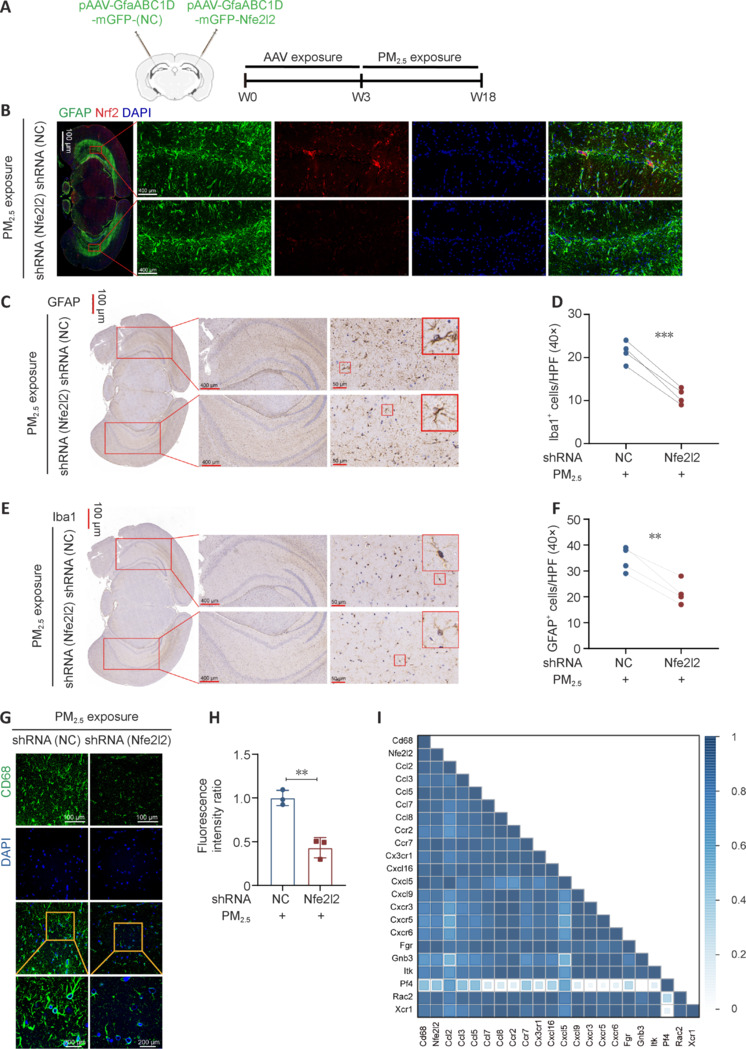
Unilateral downregulation of hippocampal nuclear factor erythroid 2-related factor 2 (NRF2) in astrocytes aggravates fine particulate matter (PM_2.5_)-induced depression-like behaviors by dampening astrocyte-to-microglia communication. (A) Schematic for the construction of unilateral *Nfe2l2* knockdown (KD) mice by the hippocampal injection of adenoviral vectors encoding short hairpin RNA (shRNA) targeting the gene encoding NRF2 (shRNA-*Nfe2l2*, *Nfe2l2* KD group) and control shRNA (shRNA-NC, NC group) in the contralateral hippocampus, followed by 15 weeks of PM_2.5_ exposure. (B) Co-expression of Nrf2 and glial fibrillary acidic protein (GFAP) in unilateral *Nfe2l2* KD mice and in the contralateral hippocampus of *Nfe2l2* NC mice after 15 weeks of PM_2.5_ exposure. Fields of view were captured at 100× magnification. (C–F) Expression of GFAP or ionized calcium binding adaptor molecule 1 (Iba1) in unilateral *Nfe2l2* KD mice and the contralateral hippocampus of mice after 15 weeks of PM_2.5_ exposure (C and E), and the numbers of GFAP^+^ (unpaired *t*-test, *t* = 3.890, *df* = 6, *P* = 0.0081, *F* = 3.675, *n* = 4) or Iba1^+^ (unpaired *t*-test, *t* = 6.622, *df* = 6, *P* = 0.0006, *F* = 1.875, *n* = 4) cells/high-power field (D and F). Scale bars: 50 μm. (n = 3 biological replicates/group). (G, H) Representative immunoreactivity (G) and quantification (H) of cluster of differentiation (CD)68 (unpaired *t*-test, *t* = 6.801, *df* = 4, *P* = 0.0024, *F* = 1.782, *n* = 4), a marker of microglial phagocytic ability, in unilateral *Nfe2l2* KD mice and in the contralateral hippocampus of NC mice after 15 weeks of PM_2.5_ exposure (*n* = 3 biological replicates/group). Fields of view were captured at 100× magnification. (I) Correlation analysis of chemokine signaling pathway-enriched genes, NRF2 and CD68, in the *Nfe2l2* KD mouse hippocampus after 15 weeks of PM_2.5_ exposure (*n* = 4 biological replicates/group). DAPI: 4′,6-Diamidino-2-phenylindole; GFAP: glial fibrillary acidic protein; Iba-1: ionized calcium-binding adapter molecule 1; KD: knockdown; NC: normal control; PM_2.5_: ambient fine particulate matter; W: week.

To clarify the specific mechanism by which astrocytic NRF2 in the hippocampus regulates PM_2.5_-induced depressive-like behaviors, we constructed a unilateral astrocytic *Nfe2l2* KD model in PM_2.5_-exposed mice through the bilateral hippocampal injection of shRNA-*Nfe2l2* (*Nfe2l2* KD group) and shRNA control (NC group) (**Figure 5A**). Immunofluorescent staining for GFAP and NRF2 was markedly reduced in the unilateral *Nfe2l2* KD mouse hippocampus compared with the contralateral hippocampus of mice after 15 weeks of PM_2.5_ exposure (**Figure 5B**), which mimics the inhibitory effect of NRF2 on hippocampal astrocyte-to-microglia communication. Similar to GFAP, the levels of Iba1 (a marker of microglial polarization) were increased in the hippocampus of *Nfe2l2* KD mice (**Figure 5C–F**), indicating that hippocampal NRF2 deficiency damages astrocyte-to-microglia communication. Furthermore, the phagocytic ability of microglia was clearly impaired, with lower microglial expression of the phagocytic marker CD68 in *Nfe2l2* KD mice than in NC mice (**Figure 5G** and **H**). Correlation analysis revealed that the mRNA level of CD68 was highly positively correlated with chemokine levels (**Figure 5I**). Collectively, these results suggest that long-term real-world PM_2.5_ exposure-induced depressive-like behaviors in mice may originate from the NRF2 deficiency-mediated disruption of astrocyte-to-microglia communication in the hippocampus.

### Targeting nuclear factor erythroid 2-related factor 2 may act as an intervention strategy for PM_2.5_-induced depression-like behaviors through remodeling astrocyte-to-microglia communication

Because NRF2 activation can play a neuroprotective role by regulating the pathological processes of neurological disorders, we investigated the protective effects of DMF and PC, a potent NRF2 agonist (Robledinos-Anton et al., 2019), on PM_2.5_-induced astrocytic A1 polarization in primary astrocytes (**Additional Figures 5** and **6**). Immunofluorescence analysis revealed that PM_2.5_-induced NRF2 was enhanced in primary astrocytes with DMF treatments (**Additional Figure 5C**). As expected, DMF significantly enhanced the protein expression of NRF2, HO-1, and NQO-1 and increased the mRNA expression of *Nfe2l2*, *Hmox1*, and *Nqo-1* following PM_2.5_ exposure (**Additional Figure 5D–J**). Additionally, PC and LBP significantly increased NRF2 protein expression (Control + Control *vs*. Control + PM_2.5_: *P* = 0.0010, Control + PC *vs.* PC + PM_2.5_: *P* = 0.0260, Control + LBP *vs.* LBP + PM_2.5_: *P* = 0.2318) and inhibited C3 protein expression (Control + Control *vs.* Control + PM_2.5_: *P* = 0.0036, Control + PC *vs*. PC + PM_2.5_: *P* = 0.0489, Control + LBP *vs.* LBP + PM_2.5_: *P* = 0.0018) in PM_2.5_-exposed astrocytes relative to control astrocytes (**Additional Figure 6D–F**). Consequently, the upregulated activation of astrocytic A1 polarization coincided with the reductions in ROS intensity, CAT, and SOD in PM_2.5_-exposed primary astrocytes upon DMF or PC treatment, indicating that NRF2 activation plays a protective role in response to PM_2.5_ challenge by inhibiting the A1 polarization of astrocytes.

**Figure 6:**
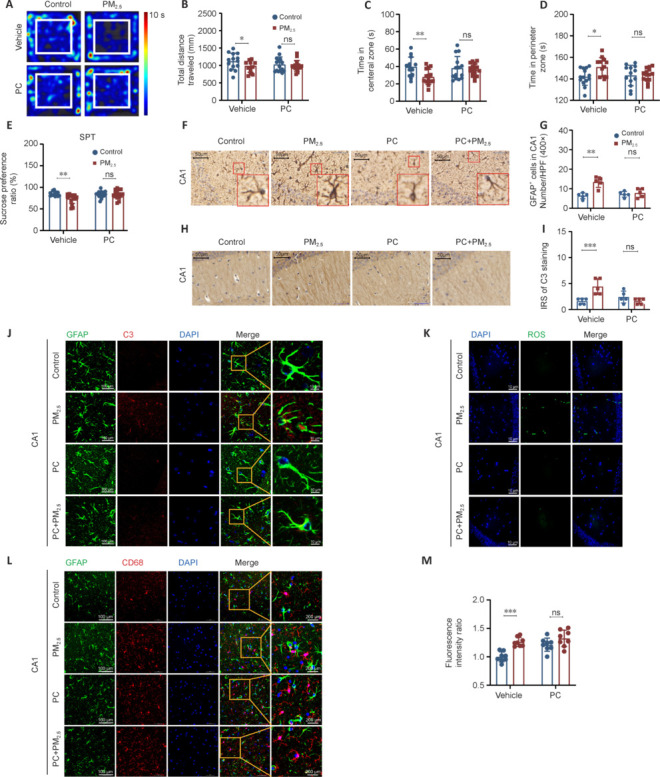
Nuclear factor erythroid 2-related factor 2 (NRF2) activation by procyanidin treatment significantly alleviates fine particulate matter (PM_2.5_)-induced depression-like behaviors through remodeling astrocyte-to-microglia communication. (A) Representative heatmaps indicating the movements of mice in the open field test (OFT) after 15 weeks of PM_2.5_ exposure, with or without procyanidin (PC) supplement. (B–D) Total distance moved (two-way ANOVA, Vehicle-Control *vs.* Vehicle-PM_2.5_: *P* = 0.0470, *n* = 13, Vehicle-PC *vs*. PM_2.5_-PC: *P* = 0.8886, *n* = 13) and time spent in the marginal (two-way ANOVA, Vehicle-Control *vs.* Vehicle-PM_2.5_: *P* = 0.0112, *n* = 13, Vehicle-PC *vs*. PC-PM_2.5_: *P* = 0.8849, *n* = 13) and central (two-way ANOVA, Vehicle-Control *vs*. Vehicle-PM_2.5_: P = 0.0131, *n* = 13, Vehicle-PC *vs.* PC-PM_2.5_: *P* = 0.9999, *n* = 13) zones of mice in the OFT after 15 weeks of PM_2.5_ exposure, with or without PC supplement (*n* = 13 biological replicates/group). (E) Sucrose consumption (two-way ANOVA, Vehicle-Control *vs.* Vehicle-PM_2.5_: *P* = 0.0069, *n* = 13, Vehicle-PC *vs*. PC-PM_2.5_: *P* = 0.9996, *n* = 13) of mice in the sucrose preference test (SPT) after 15 weeks of PM_2.5_ exposure, with or without PC supplement (*n* = 14 biological replicates/group). (F and G) Representative images of anti-glial fibrillary acidic protein (GFAP) staining in the murine cornu ammonis 1 (CA1; F) and the number of GFAP-positive cells/high-power field (400×) (G) (two-way ANOVA, Vehicle-Control *vs.* Vehicle-PM_2.5_: *P* = 0.0003, *n* = 5, Vehicle-PC *vs.* PC-PM_2.5_: *P* = 0.9666, *n* = 5) after 15 weeks of PM_2.5_ exposure, with or without PC supplement. Scale bars: 50 μm. (*n* = 5 biological replicates/group). (H, I) Representative images (H) and relative quantification (I) of anti-complement component 3 (C3) staining in the mouse CA1, and the immunoreactive score (IRS) of C3 staining (two-way ANOVA, Vehicle-Control *vs*. Vehicle-PM_2.5_: *P* = 0.0027, *n* = 5, Vehicle-PC *vs*. PC-PM_2.5_: *P* = 0.6151, *n* = 5) after 15 weeks of PM_2.5_ exposure, with or without PC supplement (*n* = 5/group, biological replicates, Mann–Whitney *U* test). Scale bars: 50 μm. (J) Co-expression of C3 and GFAP in the murine CA1 after 15 weeks of PM_2.5_ exposure, with or without PC supplement. Fields of view were captured at 100× magnification. (K) Reactive oxygen species (ROS) generation in the murine CA1 region after 15 weeks of PM_2.5_ exposure, with or without PC supplement. (L, M) Representative immunoreactive images (L) and quantification (M) of cluster of differentiation (CD)68 (two-way ANOVA, Vehicle-Control *vs*. Vehicle-PM_2.5_: *P* = 0.0003, *n* = 3, Vehicle-PC *vs*. PC-PM_2.5_: *P* = 0.2098, *n* = 3), a marker of microglia phagocytic ability, in the murine CA1 after 15 weeks of PM_2.5_ exposure, with or without PC supplement (*n* = 3 biological replicates/group). Fields of view were captured at 100× magnification. *P < 0.05, ***P < 0.001, ****P < 0.0001. ANOVA: Analysis of variance; DAPI: 4′,6-diamidino-2-phenylindole; GFAP: glial fibrillary acidic protein; IRS: immune response score; PM_2.5_: ambient fine particulate matter.

To detect the antidepressant effects of NRF2 activators, we further explored the effects of PC on real-world PM_2.5_-induced depressive-like behaviors in mice. The average concentration of PM_2.5_ was 46.48 ± 30.13 μg/m^3^ and those of its components were 2.07 ± 1.17 μg/m^3^ (BC), 10.87 ± 6.46 μg/m^3^ (OM), 7.53 ± 4.92 μg/m^3^ (NH_4_), 12.13 ± 7.85 μg/m^3^ (NO_3_), and 8.98 ± 6.44 μg/m^3^ (SO_4_) (**Additional Figure 7A**). PC treatment did not affect the body weights of mice with or without PM_2.5_ exposure (**Additional Figure 7B**). Following PM_2.5_ exposure, PC supplement effectively improved depressive-like behaviors in mice, as detected using the OFT and SPT (**Figure 6A–E**); their results were similar to those of the control mice. The immunohistochemical and immunofluorescence analyses revealed that PC treatment significantly inhibited PM_2.5_-induced astrocytic A1 polarization in the murine hippocampus through the increased expression of GFAP and C3 proteins in the CA1, CA3, and DG (**Figure 6F–J** and **Additional Figures 8–11**); these levels were comparable with those of the control/PC and PM_2.5_/PC groups. The upregulation of NRF2 activation in astrocytes ameliorated ROS generation in the hippocampal CA1, CA3, and DG of mice (**Figure 6K** and **Additional Figure 12**). Compared with control mice, CD68 protein expression was significantly higher (*P* = 0.0003) in PM_2.5_-exposed mice, and PC supplementation slightly enhanced (*P* = 0.2098) the CD68 protein level in the murine hippocampus, indicating that the phagocytic capacities of microglia may depend on NRF2 activation (**Figure 6L** and **M**). Overall, these results indicate that targeting NRF2 is predominantly protective against PM_2.5_ exposure-induced depressive-like behaviors in mice through its debilitation of hippocampus astrocytic A1 plasticity and regulation of astrocyte-to-microglia communication.

## Discussion

Depression is a common psychiatric disorder that is caused by dysfunction of the brain monoamine receptor, general monoamine system, and secondary messenger system. It is an important mental health issue and has become the 13^th^ leading cause of disability-adjusted life years worldwide (Als et al., 2023; Tan et al., 2024; Tong et al., 2024). As the main constituent of air pollution, long-term exposure to PM_2.5_ can increase the incidence risk of depression (Qiu et al., 2023; Yang et al., 2023). However, studies of the underlying mechanisms of chronic real-world PM_2.5_-induced depressive-like behaviors and potential therapeutical strategies remain scarce.

Astrocytes are vital for the synaptic plasticity of neurons; they therefore maintain the fundamental functions of neurons (Durkee et al., 2021). Our results revealed that murine depressive-like behaviors were accompanied by a shift in resting astrocytes toward the A1 phenotype during PM_2.5_ exposure. A1 astrocytes are believed to be neurotoxic and contribute to neuronal death during pathological processes by releasing toxic signals to nearby neurons (Liddelow and Barres, 2017; Liddelow et al., 2017). A1 astrocytes are characterized by increased co-expression of GFAP, C3, and S100β (Liddelow et al., 2017), similar to the alterations in astrocytes following PM_2.5_ exposure in our study. A recent investigation reported an association between A1 astrocytes and depressive-like behaviors in a murine model, which was accompanied by a loss of neurons in the hippocampus (Zhang et al., 2020). Briefly, we raised a concern of A1 astrocyte polarization during PM_2.5_ exposure in our study, which is characterized by increased GFAP, C3, S100β expression, pro-inflammation, and neurotoxic. The hippocampus is an intensively investigated brain region that is associated with depressive-like behaviors. Several studies have reported reduced hippocampal astrocyte numbers (Li et al., 2021; Writing Committee for the Attention-Deficit/Hyperactivity Disorder et al., 2021) and reduced S100β levels in patients with depression or animal models of depression. By contrast, we identified an increased number of reactive astrocytes and S100β expression in response to PM_2.5_ exposure. Interestingly, many studies support the existence of a biphasic response to stress by GFAP and S100β. For example, cerebrospinal fluid GFAP levels are significantly higher in patients with unipolar depression than in controls. Additionally, cerebrospinal fluid 100β concentrations are increased after acute predator stress (Margis et al., 2004), and circulating S100β is increased with chronic restraint stress and minor depression in male rats (Scaccianoce et al., 2004). Hence, we postulate that PM_2.5_ exposure-induced mild depressive-like behaviors resemble the effects of unpredictable chronic mild stress, which induces depressive-like behaviors and increases astrocytic reactivity (Du Preez et al., 2021).

NRF2 is a master regulator of redox balance in the CNS (Pajares et al., 2017). Moreover, astrocytic NRF2 activation exerts a protective effect on neurons by rectifying redox imbalances (Hoang et al., 2019; Wei et al., 2020; Jiwaji et al., 2022). Consistent with these previous findings, the use of DMF, an NRF2 agonist, effectively hampered A1 polarization and maintained the redox balance in astrocytes during PM_2.5_ exposure. Interestingly, PM_2.5_ exposure may lightly trigger NRF2 expression *per se*. Moreover, astrocyte–microglia–neuron interactions are driven by the astrocytic NRF2–antioxidant axis, which responds to oxidative stress and inflammation (Cuadrado et al., 2014; Silva-Palacios et al., 2018; Serapide et al., 2020). Cytokines act predominantly in an autocrine or paracrine style (Salvador et al., 2021), and some exist in the circulation and CNS, similar to hormones, and can mediate cellular interactions (DeVries et al., 2006; Deng et al., 2022).

Our findings suggest that astrocytic NRF2 deletion reduces distinct microglial and astrocytic reactivity after PM_2.5_ exposure, encompassing reduced GFAP expression, distinct morphological changes, and a weakened phagocytic ability of microglia. CD68 is a microglial marker of phagocytic activity. The efficient microglial phagocytosis of debris and damaged synapses is protective against neural impairments (Fracassi et al., 2023). This phenomenon has been observed in a chronic stress-driven depressive mouse model, in which activation of the stimulator of interferon genes pathway enhances microglial phagocytosis and ameliorates depression-like behaviors (Duan et al., 2022). These findings suggest that astrocytic NRF2 may regulate immunoregulation in microglia via cytokine-mediated cellular communication in PM_2.5_-related neurotoxicity. We therefore consider that NRF2 activation is a protective mechanism of astrocytes in response to PM_2.5_ exposure; however, this activation is insufficient to recover the aberrant regulation of astrocytes and the redox balance.

When investigating how astrocytic NRF2 signaling relates to air pollution in an attempt to improve the prevention of depression associated with PM_2.5_ pollution, many researchers have concerns about applying low-toxic chemicals to activate astrocytic NRF2, such as pyridoxine (Wei et al., 2020) and melatonin (Ali et al., 2020; Madhu et al., 2021). Here, we focused on the use of phytochemicals, which are widely used for their NRF2 activation capacities (Chun et al., 2021) and are candidates for daily dietary supplements (Qin and Hou, 2016). In our animal models, PC treatment exerted protection against PM_2.5_ exposure-induced neurotoxicity by enhancing the phagocytosis of microglia. Moreover, it did not induce notable weight loss or movement disorders. Thus, we postulate that PC may be a relatively safe candidate for depression prevention. Consistent with our findings, several studies have reported that dietary PC significantly decreases the risk of depression (Nabavi et al., 2017).

Some limitations of our study are as follows. First, we used bulk sequencing rather than single-cell sequencing methods to explore the role of NRF2 in astrocytes. Thus, the precise mechanisms underlying NRF2 in astrocytes remain to be explored. Second, the interventions were applied pre-PM_2.5_ exposure or synchronized with PM_2.5_ exposure, meaning that the reversion of depressive-like behaviors was unable to be observed in our study. Further investigations are thus required. Third, the OFT duration was relatively short. To better observe the depressive-like behaviors of mice, the duration of the OFT should be extended to 10 minutes in future studies. Notably, there is still controversy regarding the relationship between the classification of astrocytes A1 and A2 and disease progression and prognosis, which limits their application value in disease screening and treatment.

In conclusion, it is now widely recognized that air pollution is an important factor that influences mental disorders; however, the neural mechanisms through which polluted air affects mental health remain largely unknown. Our study indicated the precise mechanism of communication between two neural immune cells—namely, astrocytes and microglia—during the process of PM_2.5_ exposure-induced depression. Importantly, we also developed a therapeutic strategy to protect mice from PM_2.5_-induced neural damage and depressive-like behaviors; we targeted NRF2 signaling and upregulated NRF2 activation using PC treatment to maintain astrocyte-to-microglia communication in the hippocampus. This strategy represents a potentially economic and acceptable intervention against PM_2.5_-induced depression for public use.

## Additional files:

***Additional Table 1:*** *The primer information for quantitative reverse transcription-polymerase chain reaction assays*

***Additional Figure 1:*** *Representative immunohistochemical staining images of ionized calcium binding adaptor molecule 1 (Iba1) on murine brain sections after 15 weeks of PM2.5 exposure and control.*

Additional Figure 1 Representative immunohistochemical staining images of ionized calcium binding adaptor molecule 1 (Iba1) on murine brain sections after 15 weeks of PM_2.5_ exposure and control.

***Additional Figure 2:*** *Expression of inflammation-related genes.*

Additional Figure 2 Expression of inflammation-related genes.
(A-J) Relative expression of *Relt* (*t*=3.005, *df*=12, *P*=0.0110, *F*=2.525, *n*=8), *Mafg* (*t*=2.663, *df*=12, *P*=0.0207, *F*=2.923, *n*=8), *Jak2* (*t*=3.273, *df*=8, *P*=0.0113, *F*=1.176, *n*=8), *Nlrc5* (*t*=2.834, *df*=14, *P*=0.0133, *F*=2.368, *n*=8), *Cxcl10* (*t*=0.06355, *df* =12, *P*=0.9504, *F*=1.398, *n*=8), *Cxcr3* (*t*=0.9587, *df*=12, *P*=0.3566, *F*=1.925, *n*=8), *Stat3* (*t*=0.3682, *df*=14, *P*=0.7183, *F*=1.398, *n*=8), *NF-κB* (*t*=1.736, *df*=12, *P*=0.1082, *F*=1.166, *n*=8), *Rel* (*t*=0.2958, *df*=10, *P*=0.7735, *F*=2.057, *n*=8), *Ccl2* (*t*=0.7525, *df*=14, *P*=0.4643, *F*=1.534, *n*=8) by quantitative reverse transcription-polymerase chain reaction analysis after 15 weeks of PM2.5 exposure and control. *n* = 7-8/group, biological replicates.

***Additional Figure 3:*** *Effects of PM*_*2.5*_ *exposure on primary astrocytes.*

Additional Figure 3 Effects of PM_2.5_ exposure on primary astrocytes.
(A) Schematic diagram of extraction, culture, and identification of primary astrocytes in mice. (B) Cell Counting Kit-8 test for primarily cultured astrocytes exposed to different concentrations of PM_2.5_ (0, 5, 10, 20, 40, 80, 160 μg/mL) (*n* = 3/group, biological replicates). (C, D) Relative expression of glial fibrillary acidic protein (Gfap) (0 μg/mL *vs*. 5 μg/mL: *P*=0.9981, *n* treatment with different concentrations of PM_2.5_ exposure (0, 5, 10, and 15 μg/mL). *n* = 3/group, biological replicates. KD= kDa in E and K. (O) Co-expression of GFAP and Nrf2 in primary astrocytes after treatment with different concentrations of PM_2.5_ exposure (0, 5, 10, and 15 μg/mL). The fields of view were captured at 100 magnifications. (P, Q) Capacities of catalase (0 μg/mL *vs*. 5 μg/mL: *P*=0.1052, *n* =3, 0 μg/mL vs. 10 μg/mL: *P*=0.0096, *n* =3, 0 μg/mL *vs*. 15 μg/mL: *P*=0.0032, *n* =3) and SOD (0 μg/mL *vs*. 5 μg/mL: *P*=0.4678, *n* =3, 0 μg/mL *vs*. 10 μg/mL: *P*=0.0271, *n* =3, 0 μg/mL *vs*. 15 μg/mL: *P*=0.0035, *n* =3) in primarily cultured astrocytes after treatment with different concentrations of PM_2.5_ exposure (0, 5, 10, and 15 μg/mL). *n* = 3/group, biological replicates. (R, S) Generation of reactive oxygen species (ROS) (0 μg/mL *vs*. 5 μg/mL: *P*=0.1163, *n* =3, 0 μg/mL *vs*. 10 μg/mL: *P*=0.0345, *n* =3, 0 μg/mL *vs*. 15 μg/mL: *P*=0.0002, *n* =3) in primarily cultured astrocytes after treatment with different concentrations of PM_2.5_ exposure (0, 5, 10, and 15 μg/mL). *n* = 3/group, biological replicates.

***Additional Figure 4:*** *Nrf2 KD inhibits the expression levels of chemokines.*

Additional Figure 4 Nrf2 KD inhibits the expression levels of chemokines.
(A) The daily concentrations of ambient fine particulate matter (PM_2.5_) and its components from TAP data (sulfate [SO_4_^2−^], nitrate [NO_3_^−^], ammonium [NH4^+^], organic matter [OM], and black carbon [BC]). (B) Representative images of AAV injection GFP fluorescence expression. (C-H) Relative expression of Ccl2 (*t*=4.590, *df*=8, *P*=0.0018, *F*=58.67, *n* =6), *Ccl3* (*t* =3.005, *df*=12, *P*=0.0861, *F*=2.525, *n*=6), *Ccl5* (*t* =3.005, *df*=12, *P*=0.0110, *F* =2.525, *n*=6), *Cxcl1* (*t* =1.868, *df* =8, *p*=0.0986, *F* =7.763, *n*=6), *Cxcl2* (*t* =2.406, *df*=8, *P*=0.0428, *F*=21.82, *n*=6), *Cxcl3* (*t* =2.413, *df*=8, *P*=0.0423, *F*=22.50, *n*=6) by quantitative reverse transcription-polymerase chain reaction analysis in murine hippocampus of *Nfe2l2* knockdown (KD) and normal control (NC) group mice after 15-week PM2.5 exposure. n = 5/group, biological replicates.

***Additional Figure 5:*** *DMF-activated NRF2 expression in astrocytes prevent against A1 polarization.*

Additional Figure 5 DMF-activated NRF2 expression in astrocytes prevent against A1 polarization.
(A) Hypothesis for the role of astrocytic Nrf2 plays during fine particulate matter (PM_2.5_) exposure. (B) Cell Counting Kit- 8 test for primarily cultured astrocytes exposed to different concentrations of DMF (0, 1, 2, 3, 4, 5, 6, 7, 8, 9, and 10 μmol/mL). *n* = 3/group, biological replicates. (C) Co-expression of glial fibrillary acidic protein (GFAP) and Nrf2 in primary astrocytes after 24 hours of PM_2.5_ exposure, with or without DMF treatment. The fields of view were captured at 100×magnifications. (D-G) Relative protein expression of Nrf2 (Control:Control vs. Control:PM_2.5_: P=0.0496, *n* =3, Control:DMF vs. DMF:PM_2.5_: *P*=0.2688, *n*=3), heme oxygenase 1 (HO-1) (Control:Control *vs*. Control:PM_2.5_: *P*<0.0001, *n*=3, Control:DMF *vs*. DMF:PM_2.5_: *P*=0.0012, *n* =3), NAD(P)H dehydrogenase [quinone] 1 (NQO-1) (Control:Control *vs* Control:PM_2.5_: *P*=0.0346, *n*=3, Control:DMF vs. DMF:PM_2.5_: *P*=0.1591, *n*=3) in primary astrocytes by western blot after 24 hours PM_2.5_ exposure, with or without DMF treatment (*n* = 3/group, biological replicates). (H-J) Relative mRNA expression of Nrf2 (Control:Control *vs*. Control:PM_2.5_: *P*=0.0008, *n* =6, Control:DMF vs. DMF:PM_2.5_: *P*=0.0262, *n* =6), Ho-1 (Control:Control *vs*. Control:PM_2.5_: *P*=0.0018, *n* =6, Control:DMF *vs*. DMF:PM_2.5_: *P* = 0.0044, *n* = 6), Nqo-1 (Control:Control *vs*. Control:PM_2.5_: *P* = 0.0444, *n* = 6, Control:DMF *vs*. DMF:PM_2.5_: *P* = 0.3350, *n* = 6) in primary astrocytes by quantitative reverse transcription-polymerase chain reaction analysis after 24 hours PM_2.5_ exposure, with or without DMF treatment. *n* = 3/group, biological replicates. (M) Co-expression of GFAP and C3 in primary astrocytes after 24 hours of PM_2.5_ exposure, with or without DMF treatment. The fields of view were captured at 100 magnifications. KD= kDa in D and K. (K-L) Relative protein expression of C3 (Control:Control vs. Control:PM_2.5_: *P* = 0.0016, *n* = 3, Control:DMF VS.DMF:PM_2.5_: *P* = 0.6956, *n* = 3) in primary astrocytes by western blot after 24 hours PM_2.5_ exposure, with or without DMF treatment. *n* = 3/group, biological replicates). (N-O) Generation of reactive oxygen species (Control:Control *vs*. Control:PM_2.5_: *P* < 0.0001, *n* = 3, Control:DMF *vs*. DMF:PM_2.5_: *P* = 0.6644, *n* = 3) in primarily cultured astrocytes after 24 hours of PM_2.5_ exposure, with or without DMF treatment. (*n* = 3/group, biological replicates). (P, Q) Capacities of catalase (Control:Control *vs*. Control:PM_2.5_: *P* = 0.0023, *n* = 3, Control:DMF VS.DMF:PM_2.5_: *P* = 0.9739, *n* = 3) and superoxide dismutase (SOD) (Control:Control *vs*. Control:PM_2.5_: *P* = 0.0007, *n* = 3, Control:DMF VS.DMF:PM_2.5_: *P* = 0.9555, *n* = 3) in primarily cultured astrocytes after 24 hours of PM_2.5_ exposure, with or without DMF treatment. *n* = 3/group, biological replicates. (R) Schematic illustration for astrocytic Nrf2 activation played a protective role in PM_2.5_ treatment. DMF: Dimethyl fumarate; Nrf2/NRF2: nuclear factor erythroid 2-related factor 2.

***Additional Figure 6:*** *Phytochemicals activate NRF2 expression in astrocytes.*

Additional Figure 6 Phytochemicals activate NRF2 expression in astrocytes.
(A-C) Cell Counting Kit-8 test for primarily cultured astrocytes exposed to different concentrations of phytochemicals (procyanidin [PC], phillyrin, and lyceum barb arum polysaccharide [LBP]). *n* = 3/group, biological replicates. (D-F) Relative expression of nuclear factor erythroid 2-related factor 2 (Nrf2) (Control:Control *vs*. Control:PM_2.5_: *P* = 0.0010, *n* = 3, Control:PC vs. PC:PM_2.5_: *P* = 0.0260, *n* = 3, Control:LBP *vs*. LBP:PM_2.5_: *P* = 0.2318, *n* = 3), C3 (Control:Control *vs*. Control:PM_2.5_: *P* = 0.0036, *n* = 3, Control:PC vs. PC:PM_2.5_: *P* = 0.0489, *n* = 3, Control:LBP *vs*. LBP:PM_2.5_: *P* = 0.0018, *n* = 3) in primary astrocytes by western blot after 24 hours PM_2.5_ exposure, with or without PC/DMF treatment. *n* = 3/group, biological replicates. KD= kDa in D. (G-H) Capacities of catalase (Control:Control *vs*. Control:PM_2.5_: *P* = 0.0277, *n* = 3, Control:PC *vs*. PC:PM_2.5_: *P*=0.9999, *n* = 3) and superoxide dismutase (SOD) (Control:Control *vs*. Control:PM_2.5_: *P* = 0.0122, *n* = 3, Control:PC *vs*. PC:PM_2.5_: *P* = 0.9360, *n* = 3) in primarily cultured astrocytes after 24 hours of PM_2.5_ exposure, with or without PC/LBP treatment. *n* = 3/group, biological replicates. (I, J) ROS generation (Control:Control *vs*. Control:PM_2.5_: *P* = 0.0018, *n* = 3, Control:PC *vs*. PC:PM_2.5_: *P* = 0.9999, *n* = 3) in primarily cultured astrocytes after 24 hours of PM_2.5_ exposure, with or without PC/LBP treatment. *n* = 3/group, biological replicates. PM_2.5_: Fine particulate matter.

***Additional Figure 7:*** *Concentration of PM*_*2.5*_ *and its components.*

Additional Figure 7 Concentration of PM_2.5_ and its components.
(A) The daily concentrations of ambient PM_2.5_ and its components from TAP data (Sulfate [SO_4_^2–^], nitrate [NO_3_^–^], ammonium [NH4^+^], organic matter [OM], and black carbon [BC]). (B) The weight of mice after 15 weeks of PM_2.5_ exposure, with or without PC supplement. PM_2.5_: Fine particulate matter.

***Additional Figure 8:*** *Expression of GFAP in murine brain.*

Additional Figure 8 Expression of GFAP in murine brain.
(A-C) Representative immunohistochemical staining images of GFAP and the number of GFAP-positive cells per HPF (400×) on murine CA3 (Vehicle:Control *vs*. Vehicle:PM_2.5_: *P* = 0.0006, *n* = 5, Vehicle:PC vs. PC:PM_2.5_: *P* = 0.9999, *n* = 5) and dentate gyrus (DG) (Vehicle:Control *vs*. Vehicle:PM_2.5_: *P* = 0.0005, *n* = 5, Vehicle:PC *vs*. PC:PM_2.5_: *P* = 0.8655, *n* = 5) after 15 weeks of PM_2.5_ exposure, with or without PC supplement. ^***^P<0.001. *n* = 5/group, biological replicates. GFAP: Glial fibrillary acidic protein; HPF: high power field; PC: procyanidin; PM_2.5_: fine particulate matter.

***Additional Figure 9:*** *Expression of C3 in murine brain.*

Additional Figure 9 Expression of C3 in murine brain.
(A-C) Representative immunohistochemical staining images (A) and the immune response score (IRS) (B, C) of C3 in murine CA3 (Vehicle:Control *vs*. Vehicle:PM_2.5_: *P* = 0.041, *n* = 5, Vehicle:PC vs. PC:PM_2.5_: *P* = 0.9999, *n* = 5) and dentate gyrus (DG) (Vehicle:Control vs. Vehicle:PM_2.5_: *P* = 0.0044, *n* = 5, Vehicle:PC VS.PC:PM_2.5_: *P* = 0.9999, *n* = 5) after 15 weeks of PM_2.5_ exposure with or without PC supplement (*n* = 5/group, biological replicates). PC: Procyanidin; PM_2.5_: fine particulate matter.

***Additional Figure 10:*** *Co-expression of C3 and GFAP in murine brain after 15 weeks of PM*_*2.5*_ *exposure with or without PC supplement.*

Additional Figure 10 Co-expression of C3 and GFAP in murine brain after 15 weeks of PM_2.5_ exposure with or without PC supplement.
(A, B) Co-expression of C3 and glial fibrillary acidic protein (GFAP) in murine CA3 and dentate gyrus (DG) regions after 15 weeks of PM_2.5_ exposure, with or without PC supplement. The fields of view were captured at 100× magnifications. PC: Procyanidin; PM_2.5_: fine particulate matter.

***Additional Figure 11:*** *Co-expression of Nrf2 and GFAP in murine brain after 15 weeks of PM*_*2.5*_ *exposure with or without PC supplement.*

Additional Figure 11 Co-expression of Nrf2 and GFAP in murine brain after 15 weeks of PM_2.5_ exposure with or without PC supplement.
(A-C) Co-expression of Nrf2 and GFAP in murine CA1, CA3, and dentate gyrus (DG) regions after 15 weeks of PM_2.5_ exposure, with or without procyanidin (PC) supplement. The fields of view were captured at 100× magnifications. GFAP: Glial fibrillary acidic protein; Nrf2: nuclear factor erythroid 2-related factor 2; PM_2.5_: Fine particulate matter.

***Additional Figure 12:*** *ROS generation in murine brain after 15 weeks of PM*_*2.5*_ *exposure with or without PC supplement.*

Additional Figure 12 ROS generation in murine brain after 15 weeks of PM_2.5_ exposure with or without PC supplement.
(A-B) Generation of ROS generation in murine CA3 and dentate gyrus (DG) after 15 weeks of PM_2.5_ exposure with or without procyanidin (PC) supplement. PM_2.5_: Fine particulate matter; ROS: reactive oxygen species.

## Data Availability

*All relevant data are within the paper and its Additional files*.
