# Supplementary figures and images for: Long-term real-world PM2.5 exposure induces depression-like behaviors in mice by disrupting nuclear factor erythroid 2-related factor 2-mediated astrocyte-to-microglia communication
Source: Neural Regen Res. 2025 Aug 13;21(7):3238–48. doi: 10.4103/NRR.NRR-D-24-01469 (PMC13379045; doi:10.4103/NRR.NRR-D-24-01469)

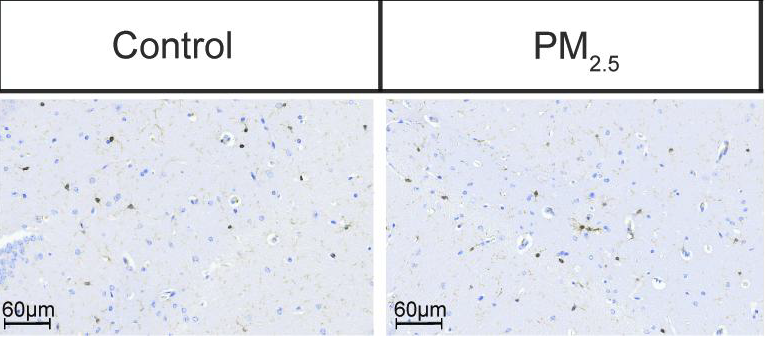

Supplement: Supplementary file 1 [file NRR-21-3238_Suppl1.tif]

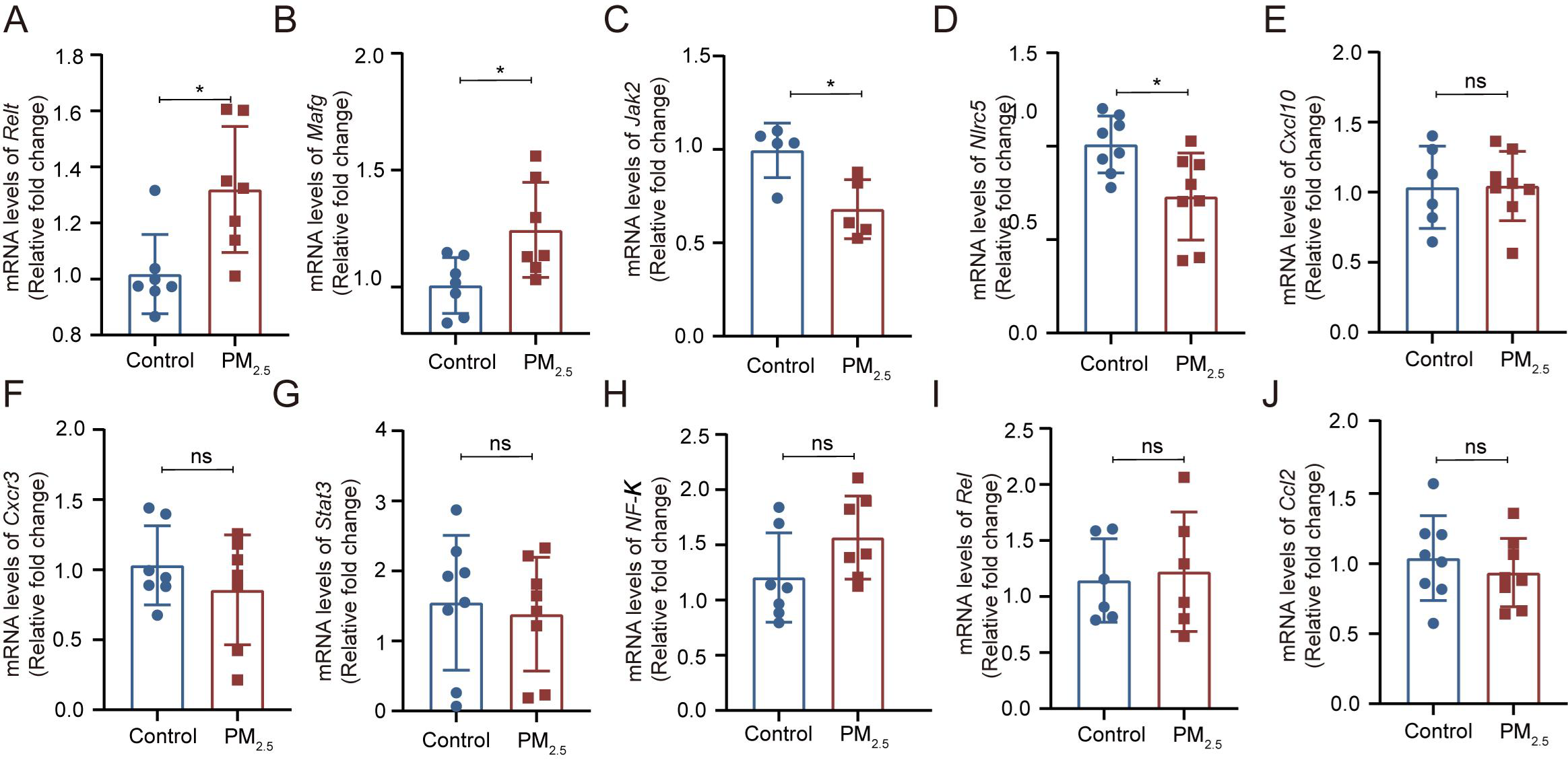

Supplement: Supplementary file 2 [file NRR-21-3238_Suppl2.tif]

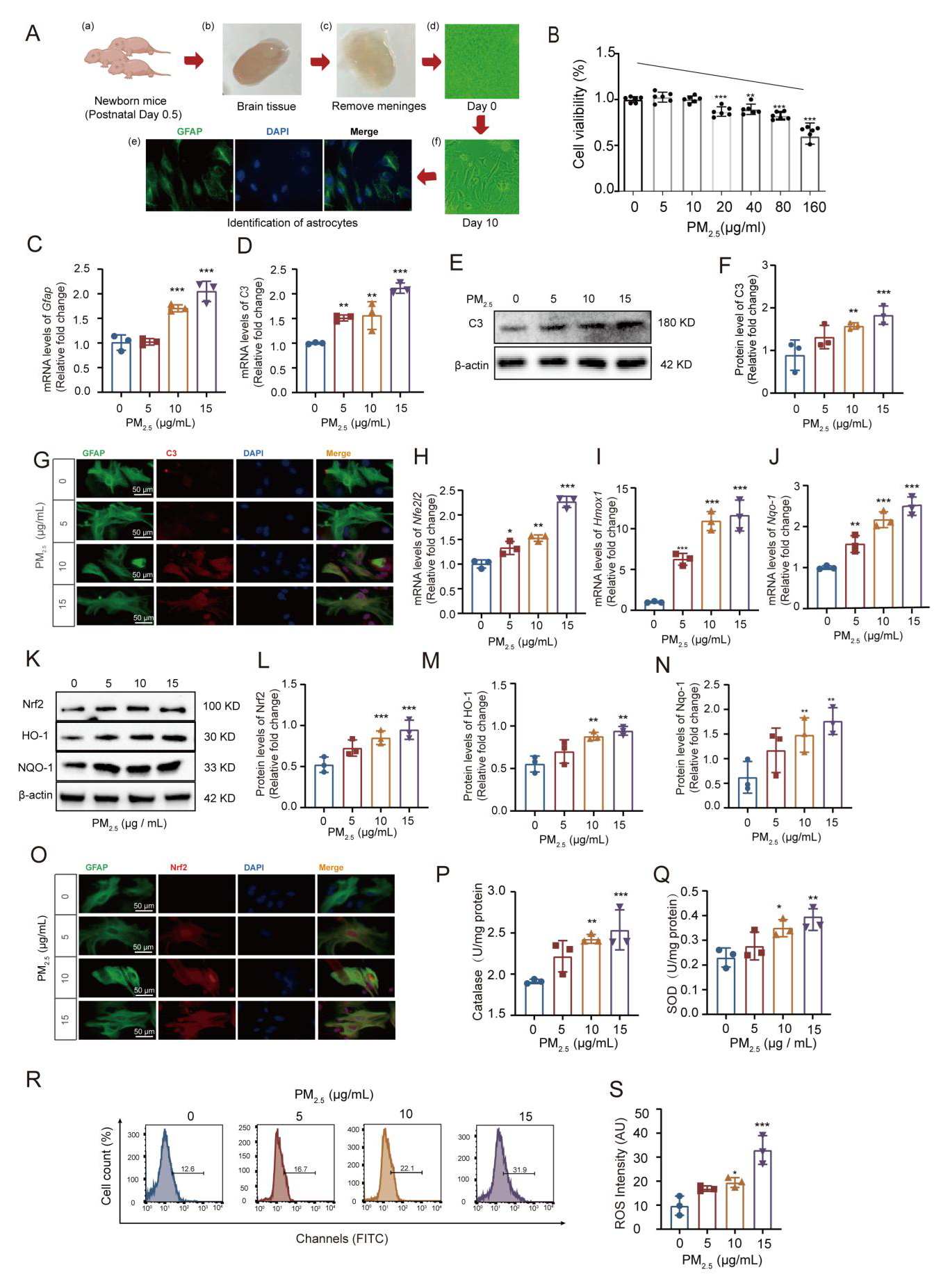

Supplement: Supplementary file 3 [file NRR-21-3238_Suppl3.tif]

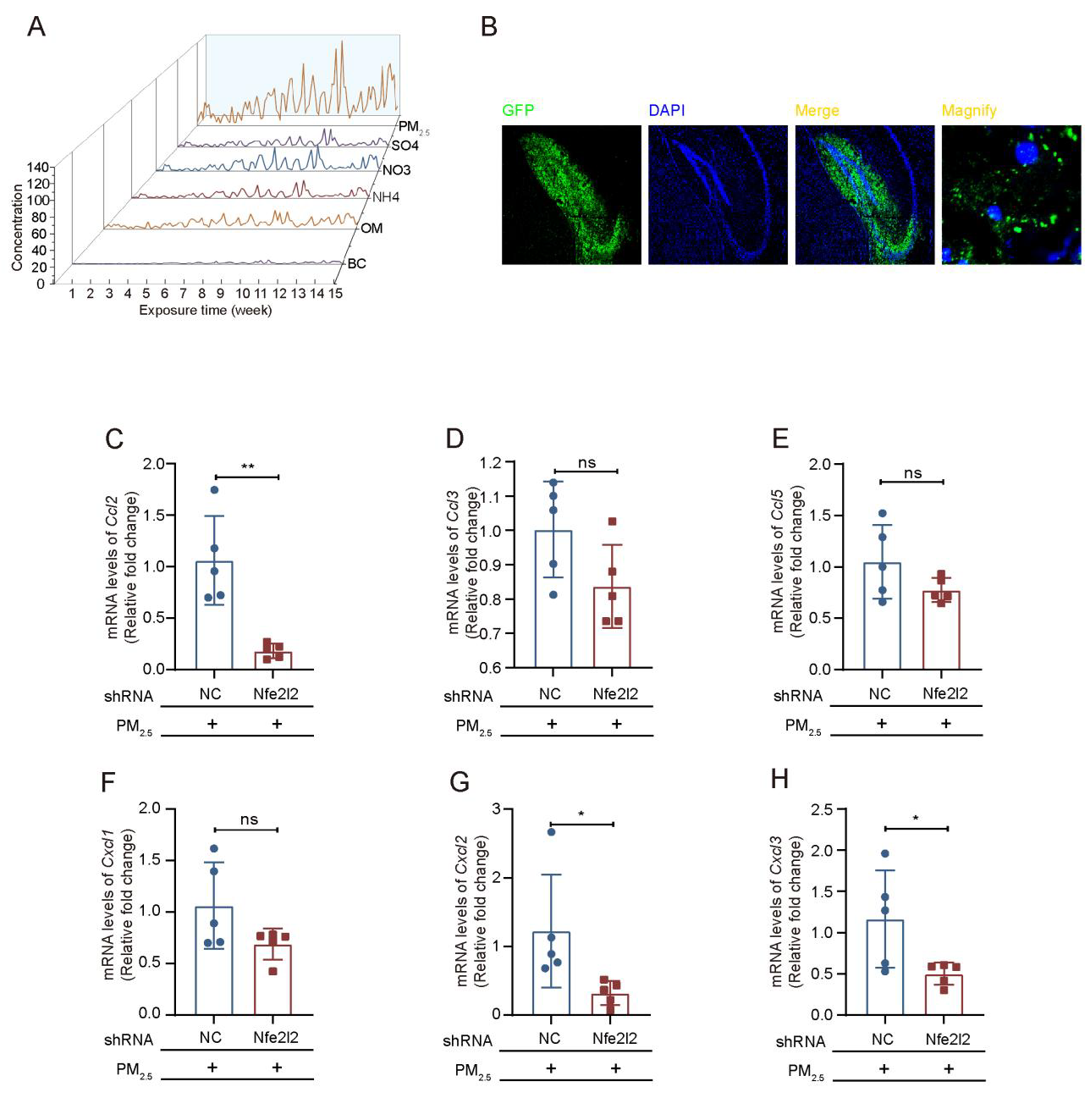

Supplement: Supplementary file 4 [file NRR-21-3238_Suppl4.tif]

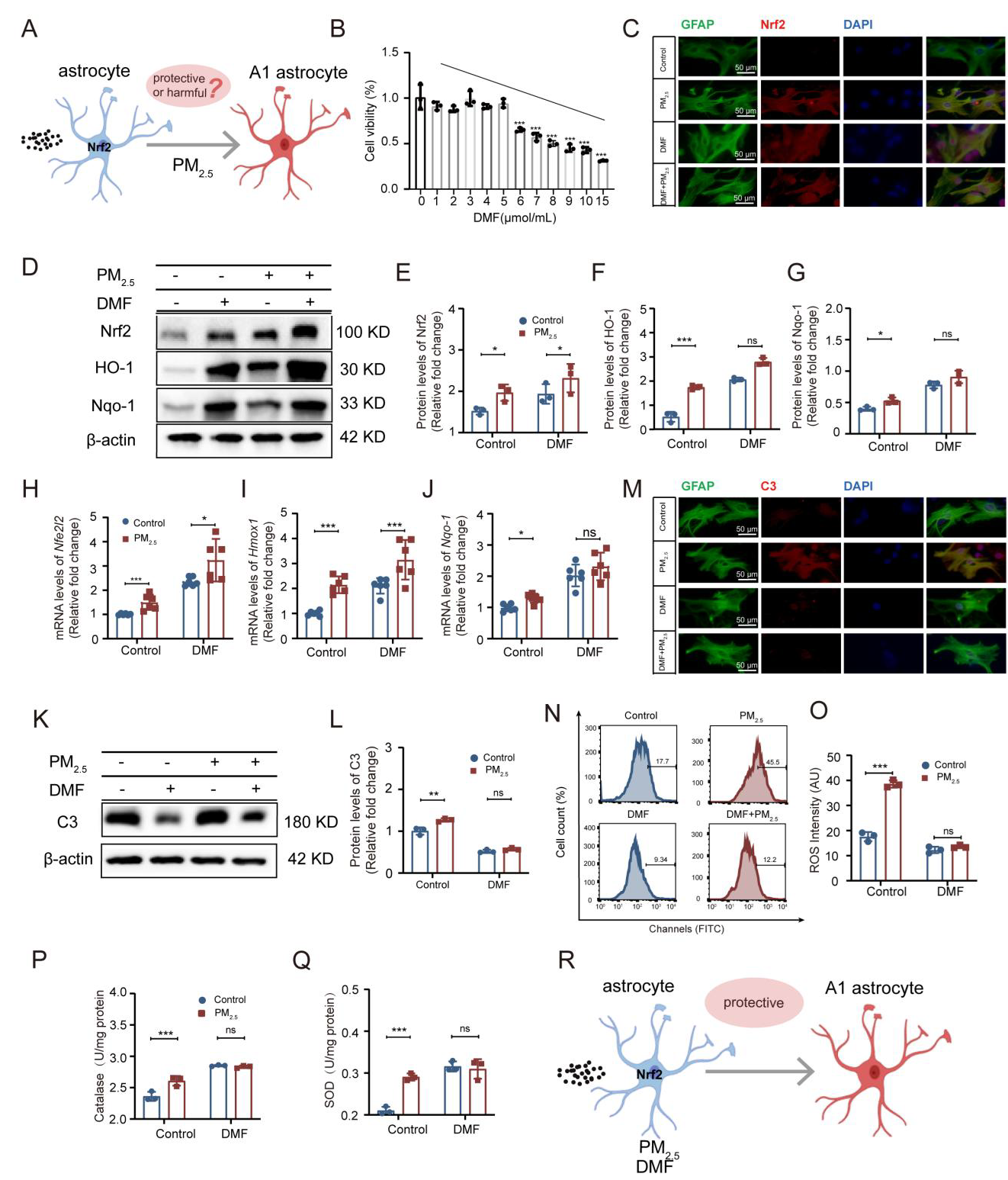

Supplement: Supplementary file 5 [file NRR-21-3238_Suppl5.tif]

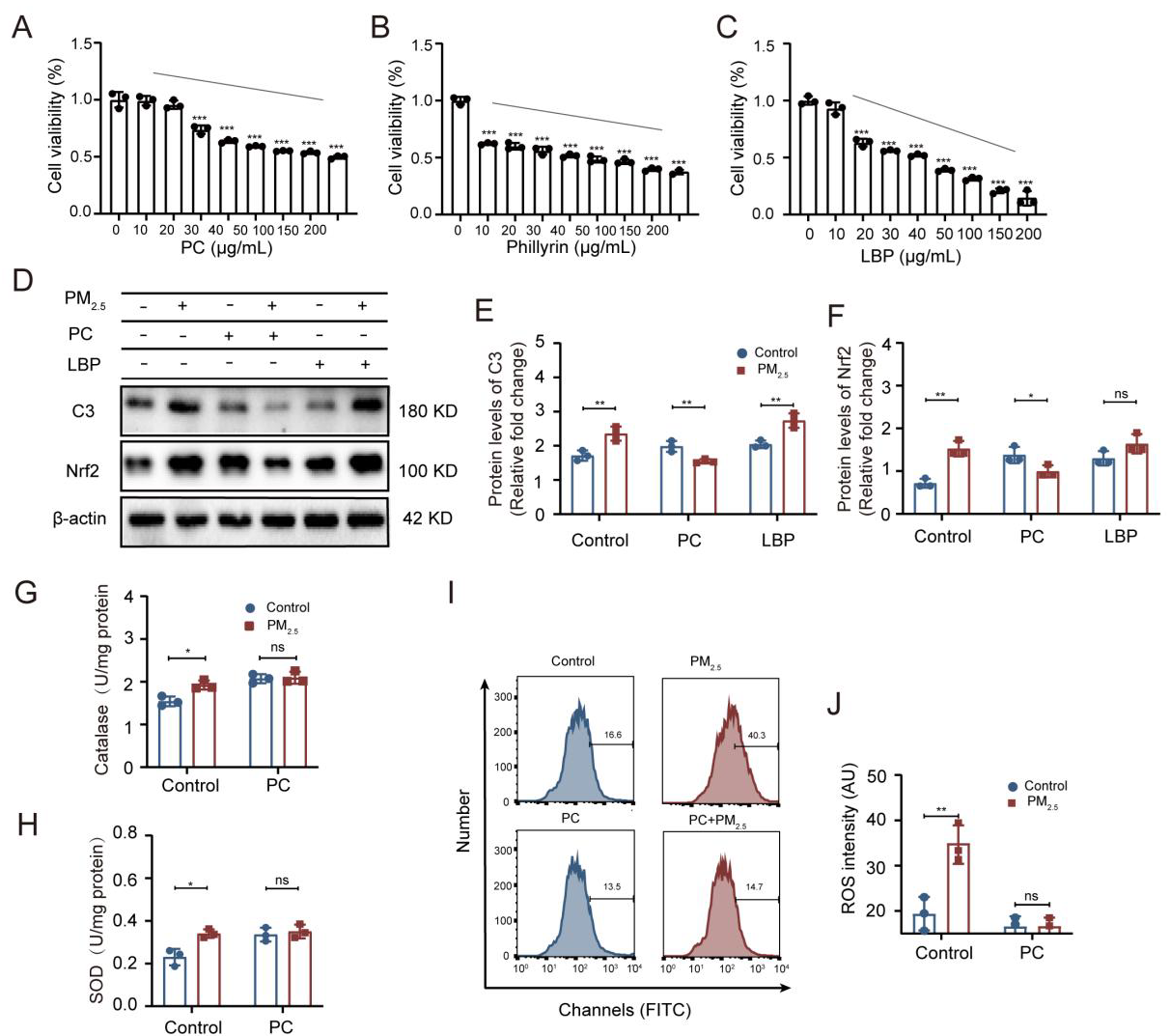

Supplement: Supplementary file 6 [file NRR-21-3238_Suppl6.tif]

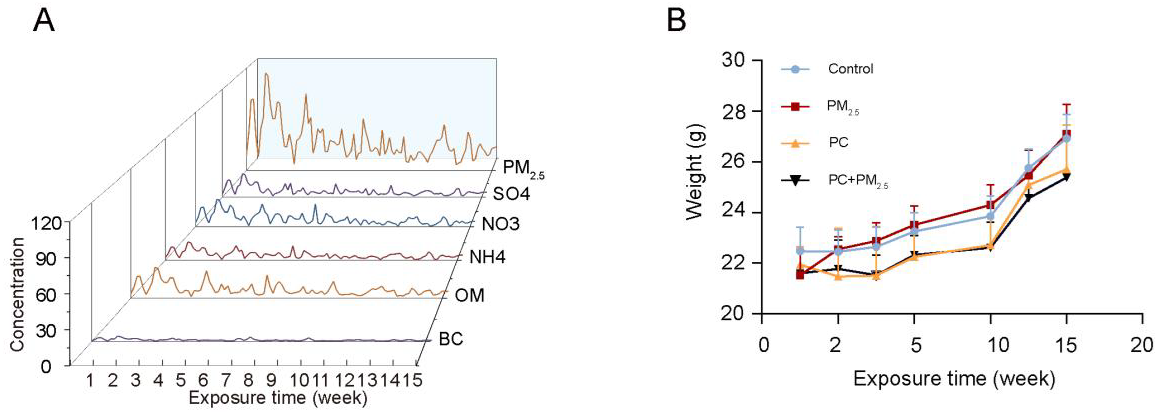

Supplement: Supplementary file 7 [file NRR-21-3238_Suppl7.tif]

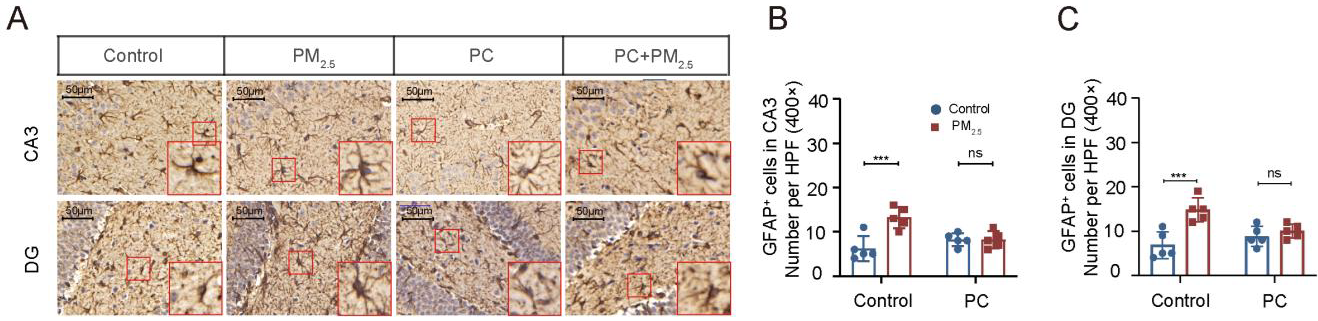

Supplement: Supplementary file 8 [file NRR-21-3238_Suppl8.tif]

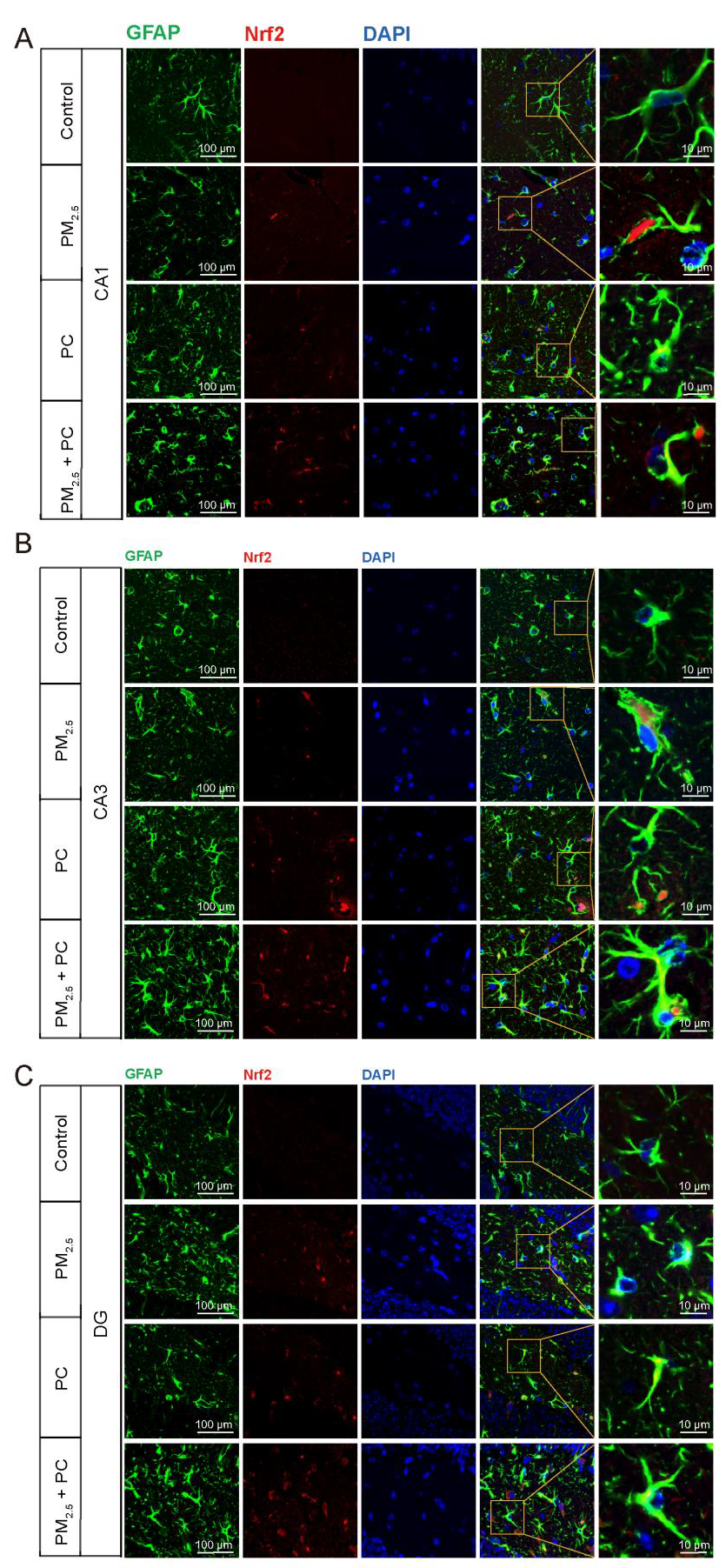

Supplement: Supplementary file 9 [file NRR-21-3238_Suppl11.tif]

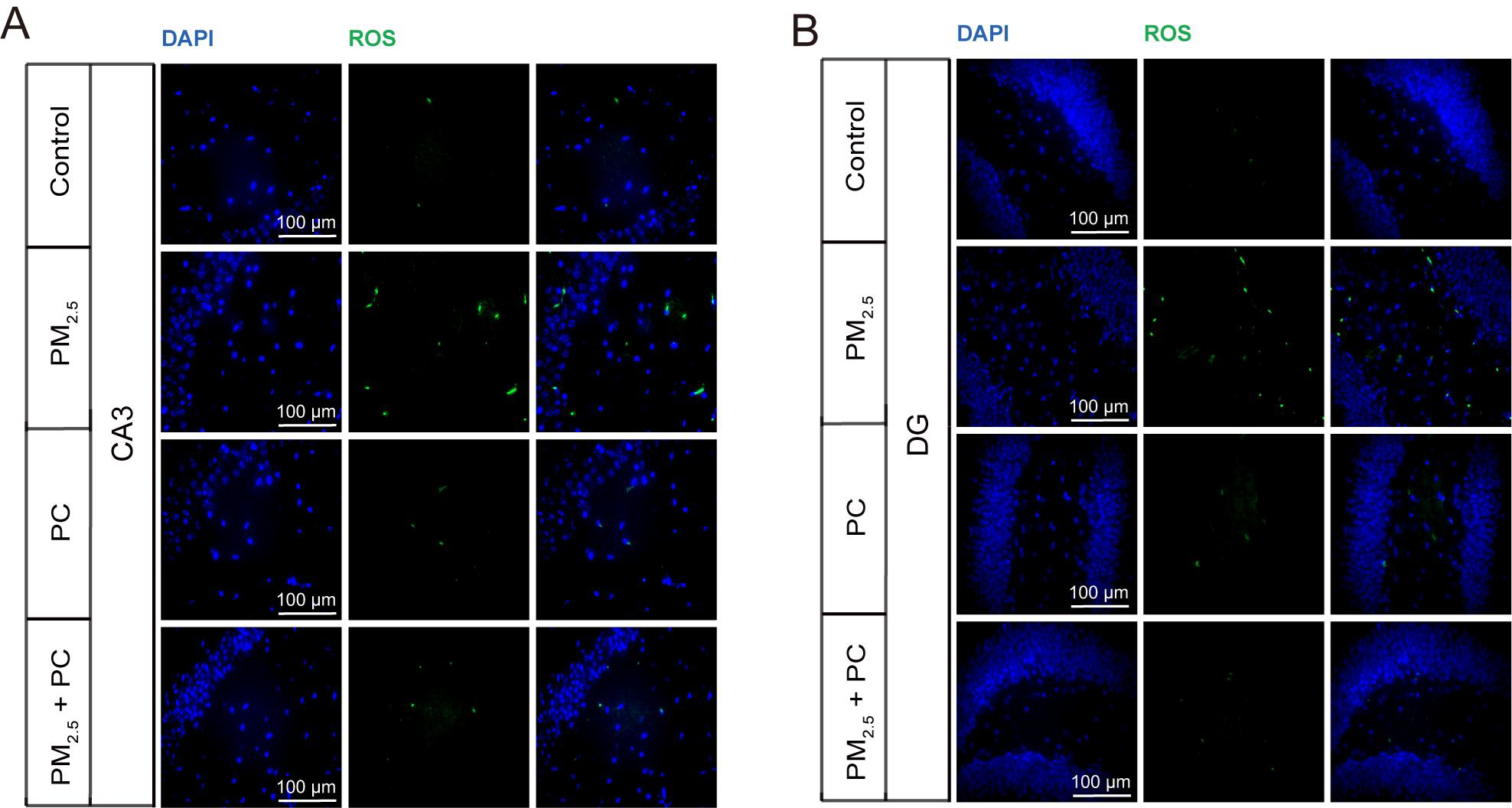

Supplement: Supplementary file 10 [file NRR-21-3238_Suppl12.tif]

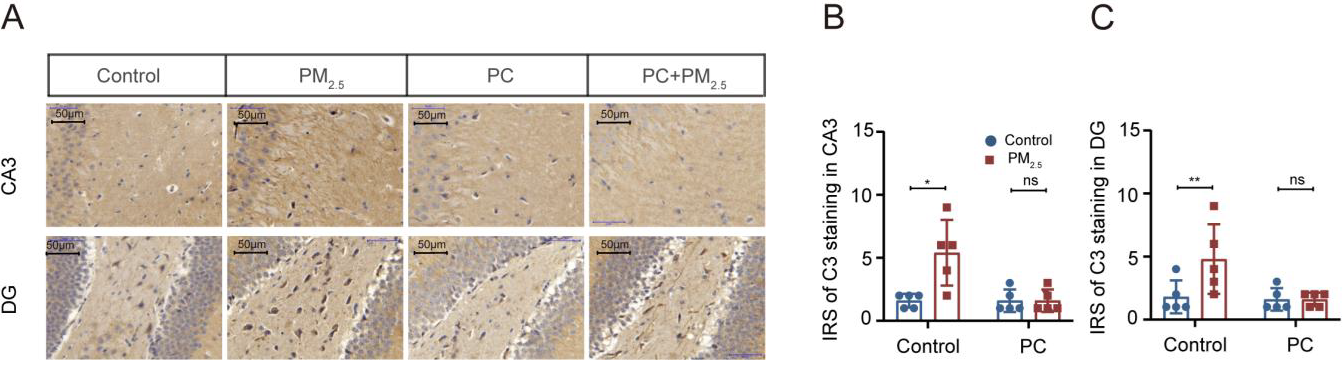

Supplement: Supplementary file 11 [file NRR-21-3238_Suppl9.tif]

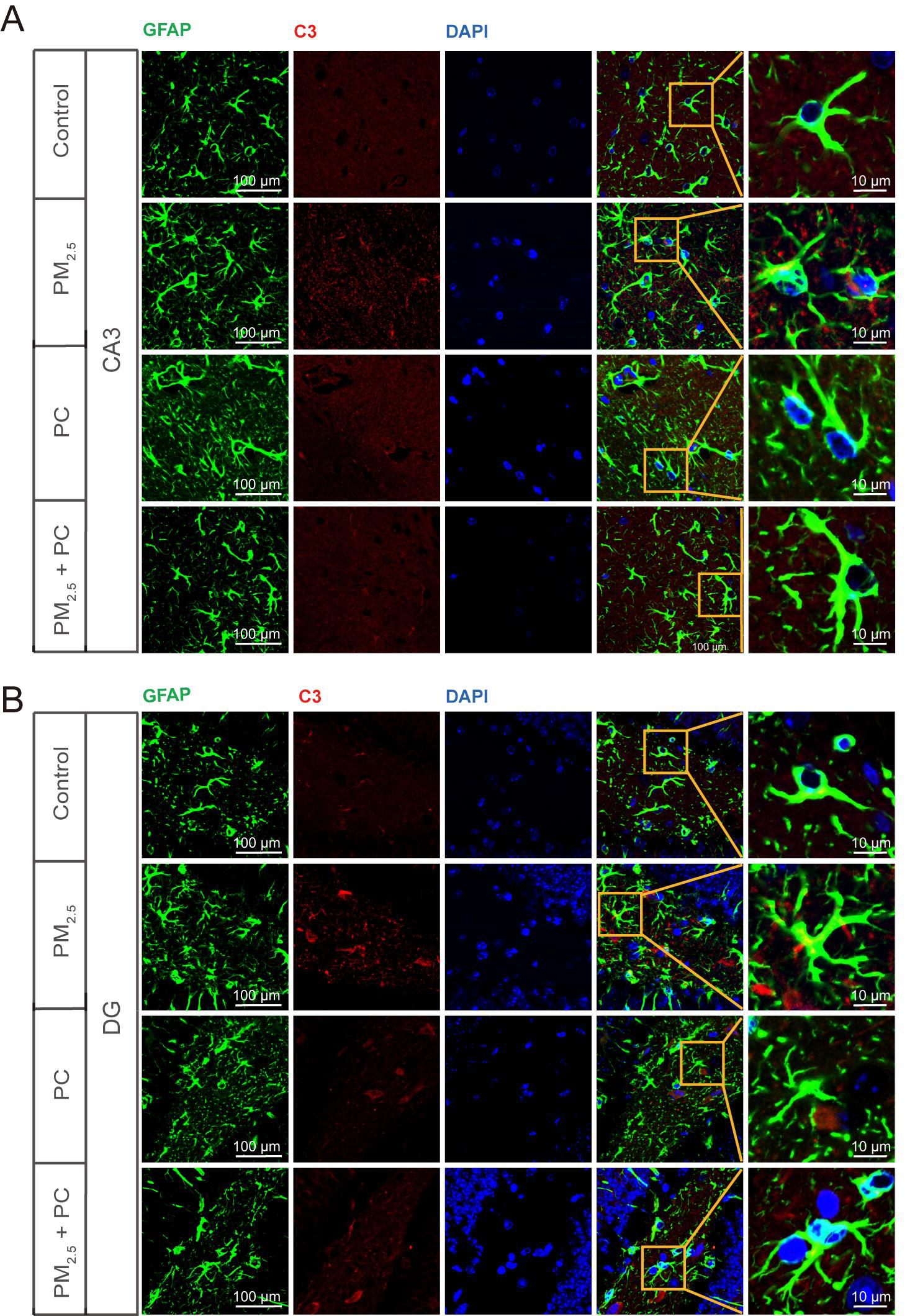

Supplement: Supplementary file 12 [file NRR-21-3238_Suppl10.tif]
